# Cyclin-Dependent Kinases 4/6 Inhibition in Glioblastoma: From Molecular Rationale to Therapeutic Challenges and Opportunities

**DOI:** 10.3390/cancers18213578

**Published:** 2026-11-06

**Authors:** Ankush Chandra, Mariana K. Najjar, Phi-Long Tran, Joshua Cha, Marc Morales, Hui-Wen Lo

**Affiliations:** 1 Vivian L. Smith Department of Neurosurgery, McGovern Medical School, The University of Texas Health Houston, Houston, TX 77030, USA; mariana.k.najjar@uth.tmc.edu (M.K.N.); philongtran@tamu.edu (P.-L.T.); joshua.cha@tamu.edu (J.C.); mormarc99@gmail.com (M.M.); hui-wen.lo@tamu.edu (H.-W.L.); 2 Department of Cell Biology and Genetics, College of Medicine, Texas A&M University, College Station, TX 77843, USA; 3 Graduate School of Biomedical Sciences, The University of Texas Health Science Center at Houston, Houston, TX 77030, USA; 4 Department of Neurology, Virginia Tech Carilion, Roanoke, VA 24016, USA

**Keywords:** cyclin-dependent kinases, CDK4, CDK6, glioblastoma, CDK4/6 inhibitor, targeted therapy, combination therapy

## Abstract

Glioblastoma (GBM) remains the most lethal primary malignant brain tumor in adults despite maximal resection, radiotherapy, and temozolomide. The cyclin D-CDK4/6-RB axis plays a significant role in cell cycle regulation and is amongst the most frequently dysregulated pathways in many cancers including GBM, thus providing a strong rationale for using CDK4/6 inhibitors (CDK4/6is) in treating this tumor. While palbociclib, ribociclib, and abemaciclib are FDA-approved to treat hormone positive breast cancer, they have demonstrated robust target engagement and efficacy in preclinical studies in GBM. However, clinical trial data has been modest, highlighting a major gap between biological rationale and therapeutic efficacy in the clinical setting. In this comprehensive review, we examine the molecular functions of CDK4/6 in GBM, delve into the pharmacologic properties of approved and emerging inhibitors, explore mechanisms of drug resistance and discuss combination strategies, clinical experience till date, and potential biomarkers for ideal patient selection. Future progress will likely require CNS-active inhibitors, rational combination therapies, functional biomarker selection, and early confirmation of adequate tumor drug exposure and pathway suppression.

## Simple Summary

Glioblastoma (GBM) is an aggressive brain tumor with limited treatment options and poor outcomes. CDK4 and CDK6 are key regulators of cell division and are frequently dysregulated in glioblastoma, making them attractive therapeutic targets. Although CDK4/6 inhibitors have shown activity in laboratory models, clinical benefit in GBM patients has remained limited. This review examines the biological basis for targeting CDK4/6 in GBM, the challenges of drug delivery to the brain, mechanisms of treatment resistance, and the current clinical experience with these agents. We also discuss emerging combination strategies, brain-penetrant inhibitors, and biomarkers that may help identify patients most likely to benefit. Better integration of tumor biology, drug exposure, and patient selection may improve the future development of CDK4/6-directed therapy in glioblastoma.

## 1. Introduction

Glioblastoma (GBM) is the most common and aggressive adult brain malignancy, accounting for more than half of all primary malignant gliomas. Under the most recent 2021 World Health Organization (WHO) Classification of Central Nervous System (CNS) Tumors, GBM is defined as an IDH-wildtype tumor with characteristic histologic and/or molecular features [1]. According to the most recent Central Brain Tumor Registry of the United States (CBTRUS) Statistical Report in 2025, the average annual age-adjusted incidence rate of all malignant and non-malignant brain and other CNS tumors was 25.34 per 100,000 people between 2017 and 2021 [2]. Among these, GBM was the most commonly occurring malignant brain and other CNS tumor, representing 13.7% of all tumors and 52.2% of malignant tumors. The prognosis of GBM remains very poor, with a five-year relative survival rate of only 7.1% [3].

Despite significant advances in biomedical sciences and our understanding of GBM biology, the median overall survival (OS) for patients remains dismal at 12–16 months [4]. The current standard of care for newly diagnosed GBM consists of maximal safe surgical resection followed by radiotherapy (RT) with concurrent and adjuvant temozolomide (TMZ), called the Stupp protocol, that has not changed since 2005 [5]. The landmark EORTC-NCIC trial established this approach, demonstrating an improvement in median OS from 12.1 months with radiotherapy alone to 14.6 months with radiotherapy and TMZ, as well as an increase in 2-year survival from 10.4% to 26.5% [5]. A subsequent follow-up study confirmed the durability of this survival benefit [6]. Unfortunately, nearly all GBMs ultimately recur and progress. This treatment failure can be attributed to several characteristics of the disease, including diffuse infiltration into surrounding parenchyma, profound intratumoral and intertumoral heterogeneity, treatment-resistant cellular populations, glioma stem cell renewal, activation of DNA damage repair and survival pathways, and restricted delivery of many systemic agents across the blood–brain (BBB) and blood–tumor barriers (BTB) [7,8]. These limitations have driven continued efforts to identify molecular vulnerabilities that can be therapeutically exploited for greater success.

**Figure 1:**
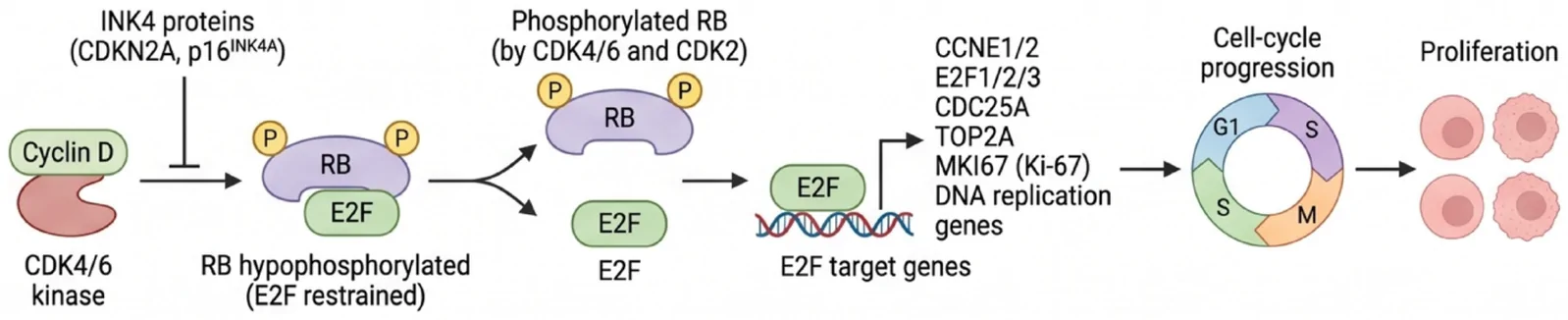
Canonical CDK4/6–RB–E2F signaling pathway regulating cell-cycle progression. Cyclin D-activated CDK4/6 phosphorylates the RB protein, relieving RB-mediated sequestration of E2F transcription factors. The release of E2F promotes transcription of genes required for G1/S transition and DNA replication, which include CCNE1/2, E2F1/2/3, CDC25A, TOP2A, and MKI67 (Ki-67), thereby driving cell-cycle progression and proliferation. INK4 family proteins, including CDKN2A/p16INK4A, inhibit CDK4/6 activity and maintain RB in its hypophosphorylated, growth-suppressive state, thereby acting as natural breaks. Further RB phosphorylation by CDK4/6 and CDK2 reinforces E2F activation and commitment to S-phase entry. Created in BioRender. Najjar, MK (2026). https://BioRender.com/qu7zyec.

Cell cycle control dysregulation represents one of the most frequent molecular abnormalities in GBM. Large-scale genomic analyses have identified recurrent alterations involving the receptor tyrosine kinase (RTK)/RAS/phosphoinositide 3-kinase, p53, and retinoblastoma (RB) signaling networks, with disruption of the RB pathway occurring in the majority of tumors [9,10]. A central component of this pathway is the cyclin D-cyclin-dependent kinase 4/6 (CDK4/6)-RB axis. Under physiologic conditions, mitogenic signaling promotes formation of cyclin D-CDK4 and cyclin D-CDK6 complexes, which phosphorylate RB and release E2F transcription factors, thereby permitting progression through the G1/S cell-cycle checkpoint. Conversely, endogenous CDK inhibitors such as p16INK4A restrain this process. In GBM, this regulatory system is frequently disrupted through alterations including CDKN2A/B deletion, CDK4 amplification, CDK6 dysregulation, and RB1 loss, collectively promoting uncontrolled cell-cycle progression (Figure 1) [9,10,11].

These genomic alterations provide a strong biological rationale for targeting CDK4/6 in GBM. Preclinical studies have demonstrated that pharmacologic CDK4/6 inhibition can induce G1 arrest and suppress the growth of RB-intact GBM models, with response influenced by the molecular status of the p16-CDK4-RB pathway [11,12]. Moreover, the biological functions of CDK4 and CDK6 may extend beyond canonical cell-cycle regulation and intersect with pathways governing DNA damage responses, cellular plasticity, invasion, metabolism, and treatment resistance. These observations, together with the substantial clinical success of selective CDK4/6 inhibitors in other malignancies such as breast cancer, have sparked interest in translating this therapeutic strategy to GBM.

While preclinical studies showed promising results, clinical trials in GBM have thus far failed to translate the preclinical promise into significant survival benefits for patients. For instance, palbociclib demonstrated limited activity as monotherapy in patients with recurrent RB1-positive GBM [13], while studies of ribociclib demonstrated measurable intratumoral drug exposure and pharmacodynamic target inhibition without durable clinical benefit [14]. These findings suggest that the translational gap might be due to several mechanisms such as heterogeneous CNS drug exposure, adaptive signaling, tumor-cell plasticity, and insufficient biomarker selection, to name a few. Moreover, drug-specific variability in CNS penetration further underscores this challenge [15].

Thus, the central question is no longer simply whether CDK4/6 represents a biologically relevant target in GBM, but why inhibition of this highly dysregulated pathway has failed to produce meaningful clinical efficacy and how this therapeutic vulnerability can be more effectively exploited. In this review, we examine the molecular and functional roles of CDK4 and CDK6 in GBM, the pharmacologic characteristics and CNS penetrance of available and emerging CDK4/6 inhibitors, and the preclinical and clinical experience with these agents. Additionally, particular emphasis is placed on mechanisms of intrinsic and adaptive resistance, rational combination strategies, and biomarkers that may enable more precise patient selection. Understanding the disconnect between compelling molecular rationale and limited clinical efficacy will be essential for defining the future role of CDK4/6-directed therapy in GBM.

## 2. The CDK4/6-Cyclin D-Rb Axis in Glioblastoma

### 2.1. Canonical CDK4/6-Rb Cell-Cycle Signaling

The cyclin D-CDK4/6-RB pathway is a central gatekeeper of the G1-to-S cell-cycle transition and remains one of the most frequently disrupted regulatory systems in GBM. Normally, in quiescent cells, hypophosphorylated RB binds E2F transcription factors and restrains expression of genes required for DNA synthesis and S-phase entry. Mitogenic signaling induces D-type cyclins (cyclin D1/CCND1, D2/CCND2, and D3/CCND3), which assemble with CDK4 and CDK6. The resulting kinase complexes phosphorylate RB, thereby weakening RB-E2F binding, and permitting E2F-dependent transcription of genes that drive DNA replication and cell-cycle progression. This canonical sequence provides the mechanistic basis for targeting CDK4/6 in tumors that retain functional RB [9,16].

The principal endogenous brakes on this pathway are members of the INK4 and CIP/KIP families. In particular, p16INK4A, encoded by CDKN2A, binds CDK4/6 and prevents cyclin D-CDK4/6 complex formation. p15INK4B (CDKN2B) and p18INK4C (CDKN2C) provide related inhibitory functions, while p21CIP1 and p27KIP1 modulate cyclin-CDK complex assembly and activity. Thus, loss of CDKN2A/B removes a major inhibitory checkpoint and can render cell-cycle progression increasingly dependent on CDK4/6 activity. On the contrary, loss or functional inactivation of RB1 eliminates the downstream brake that CDK4/6 inhibitors are designed to restore. Hence, this explains why intact RB is a fundamental determinant of biological response to CDK4/6 blockade in GBM models [11,12].

**Figure 2:**
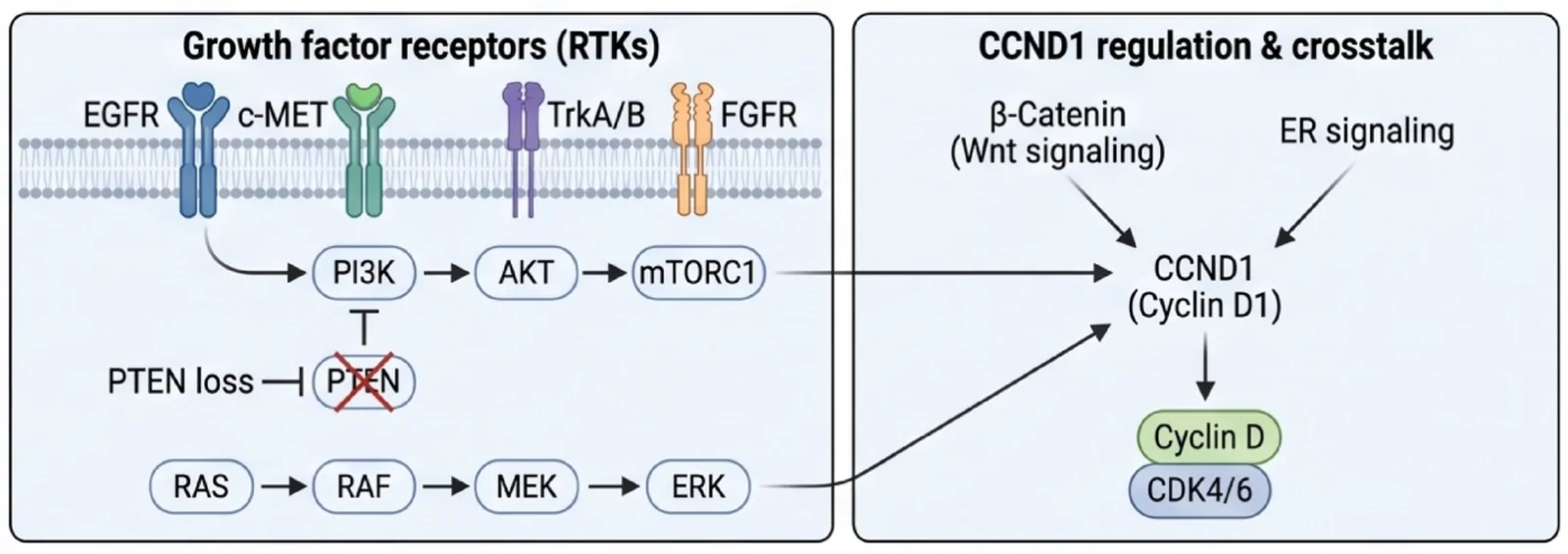
Upstream signaling and crosstalk regulating the Cyclin D–CDK4/6 axis. Growth factor receptor tyrosine kinases, including EGFR, c-MET, TrkA/B, and FGFR, activate downstream signaling through the PI3K-AKT-mTORC1 and RAS-RAF-MEK-ERK pathways, promoting CCND1 (Cyclin D1) expression and CDK4/6 activation. Loss of the tumor suppressor PTEN further enhances PI3K-AKT signaling by relieving negative regulation of the pathway. Additional signaling inputs, including Wnt/β-catenin and estrogen receptor (ER) signaling, converge on CCND1 regulation. Together, these upstream pathways integrate mitogenic and oncogenic signals to modulate Cyclin D-CDK4/6 activity and cell-cycle progression. Created in BioRender. Najjar, MK (2026). https://BioRender.com/qu7zyec.

It is important to understand that this pathway is not an isolated linear module. In GBM, RTK signaling, notably EGFR and PDGFRA, feeds into RAS-RAF-MEK-ERK and PI3K-AKT-mTOR pathways that converge on cyclin D expression, stability, and activity. ERK signaling promotes cyclin D transcription through AP-1-dependent programs, whereas AKT signaling can enhance cyclin D activity through several mechanisms, including inhibition of GSK3β-mediated cyclin D turnover. This places CDK4/6 as a downstream integrator of oncogenic growth signals rather than simply a housekeeping cell-cycle kinase (Figure 2). This signaling coupling is further supported by patient-derived GBM xenograft models, as shown by Zhang et al. [17].

### 2.2. Genomic Dysregulation of the Pathway in GBM

The molecular basis for targeting CDK4/6 in GBM was established long before selective CDK4/6 inhibitors entered clinical practice. Early cytogenetic and molecular studies of malignant gliomas identified recurrent amplification of chromosome 12q13-14, with CDK4 among the most consistently amplified genes [18]. Schmidt and colleagues subsequently demonstrated that CDKN2A deletion or CDK4 amplification occurred in the majority of GBMs in their cohort, establishing two alternative mechanisms for disabling the same G1 checkpoint [19]. Additional studies showed that CDK4 amplification is accompanied by increased CDK4 protein expression, supporting its functional role rather than merely a passenger role [20]. CDK6 dysregulation is less commonly driven by focal amplification, but elevated CDK6 expression has been documented in GBM relative to matched normal brain, suggesting that both kinases can contribute to pathway activation [21]. Immunohistochemical and molecular analyses further showed that alterations involving CDKN2A, CDK4, and RB1 commonly converge on disruption of the p16-CDK4-Rb cascade rather than representing unrelated events [22].

**Figure 3:**
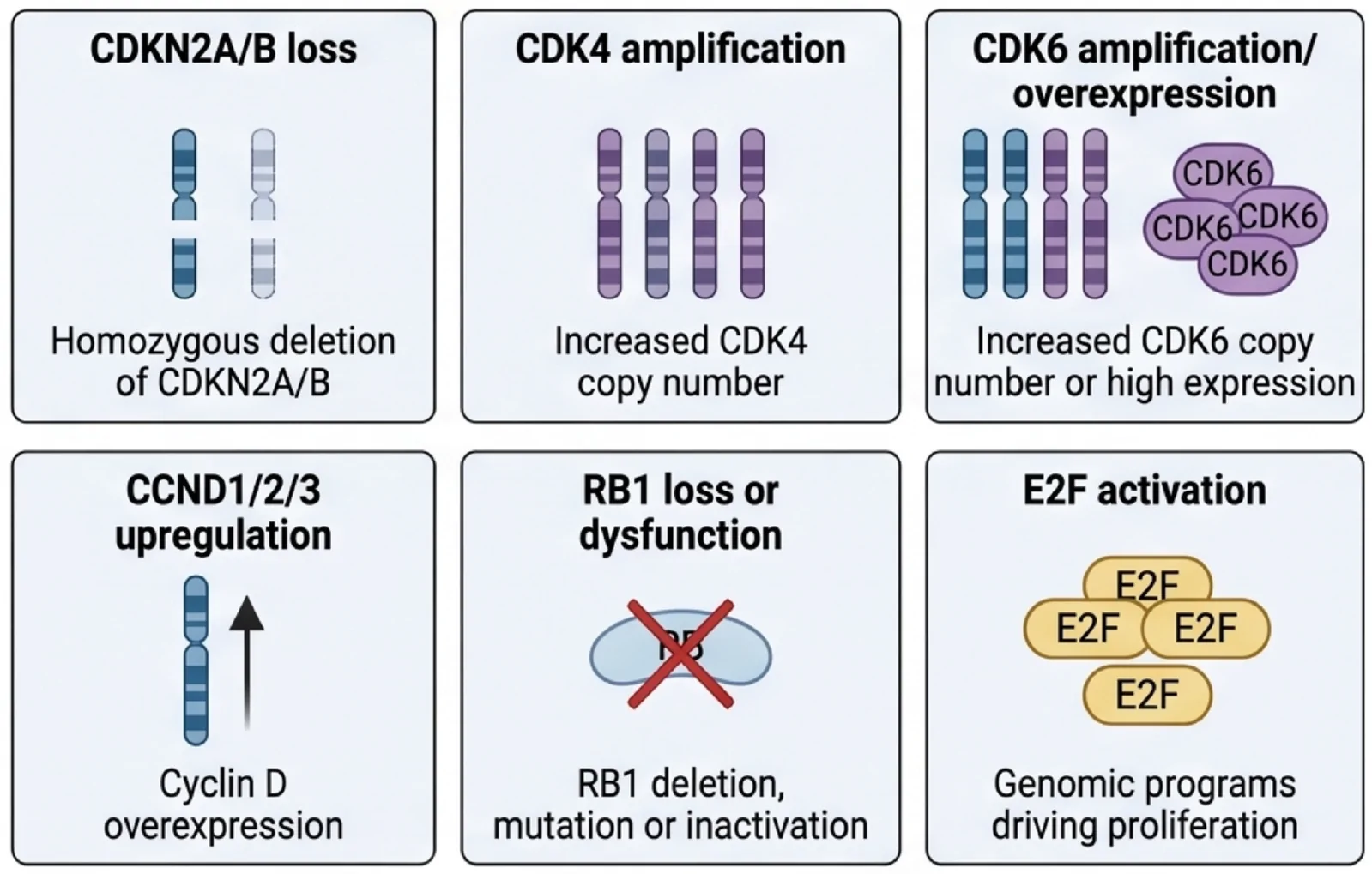
Recurrent alterations of the CDK4/6-RB-E2F pathway in glioblastoma. Glioblastoma frequently harbors genomic and transcriptional alterations that converge on dysregulation of the CDK4/6-RB-E2F axis. These include homozygous deletion of CDKN2A/B, CDK4 amplification, CDK6 amplification or overexpression, and upregulation of CCND1/2/3, all of which promote Cyclin D-CDK4/6 pathway activity. In contrast, RB1 deletion, mutation, or functional inactivation disrupts the principal downstream tumor-suppressive checkpoint and may confer relative insensitivity to CDK4/6 inhibition. Collectively, these alterations facilitate E2F activation and induction of proliferative transcriptional programs that drive cell-cycle progression in glioblastoma. Created in BioRender. Najjar, MK (2026). https://BioRender.com/qu7zyec.

Large-scale genomic studies have since placed these observations into a broader framework. The Cancer Genome Atlas (TCGA) demonstrated that the RB pathway is one of three core signaling networks recurrently altered in GBM, alongside RTK/PI3K and p53 [9]. Subsequent analysis of over 500 tumors by Brennan et al. confirmed that alterations affecting CDKN2A/B, CDK4, CDK6, CCND genes, and RB1 collectively constitute a dominant genomic feature of IDH-wildtype GBM [10]. Interestingly, certain alterations are often functionally redundant, namely, homozygous CDKN2A/B deletion, CDK4 amplification, CDK6 activation, and RB1 loss, with each being able to deregulate the G1/S checkpoint (Figure 3). However, it is important to know that they do not necessarily predict identical therapeutic responses. For example, a tumor with CDKN2A loss but intact RB may remain dependent on CDK4/6, whereas RB1-deficient tumors are intrinsically less likely to respond to selective CDK4/6 inhibition [11,12].

One important distinction is that the genomic alteration of the CDK4/6-RB pathway is not synonymous with functional dependence on CDK4/6. Functional dependency implies that continued tumor proliferation remains contingent on CDK4/6 mediated RB phosphorylation, such that pharmacologic blockade produces sustained suppression of RB-E2F signaling. Important to note is that in GBM, RB competence is a prerequisite such that RB-deficient models are intrinsically resistant to CDK4/6 inhibition, while RB-proficient models are able to undergo profound G1 arrest [12]. However, intact RB alone is insufficient, as exemplified by an RB1-positive recurrent GBM trial of palbociclib that demonstrated minimal clinical activity despite molecular selection [13]. Upstream genomic dysregulations such as CDKN2A/B loss or CDK4/6 amplification indicate pathway activation but do not necessarily establish therapeutic addiction. This is demonstrated in the work by Cen et al., where patient-derived models showed greatest palbociclib sensitivity in CDKN2A/B-deleted, RB-intact tumors, whereas some CDK4-amplified, p16-retaining models remained resistant [11]. Intratumoral heterogeneity further complicates this relationship because genetically distinct subclones and plastic cellular states coexist within individual GBMs and may differ in CDK4/6 dependence, as further discussed below [23]. Consistent with this, Li et al.’s work showed that proneural glioma stem-like cells with intact RB and higher CDK6 expression were preferentially sensitive to CDK4/6 inhibition, yet treatment promoted a shift toward mesenchymal-associated programs [24]. Thus, pathway alteration should be viewed as evidence of pathway activation, whereas true therapeutic dependency is more likely to require functional RB, demonstrable suppression of RB-E2F output, and absence of dominant resistant subpopulations or bypass states.

Older literature on amplification also anticipated the complex genomic architecture now recognized in GBM, as shown by Furgason et al. This study showed that CDK4 frequently resides within broad or complex amplicons that may include MDM2, and chromothripsis can generate co-amplification of oncogenic loci including EGFR, MDM2/MDM4, and CDK4 [25]. Thus, CDK4 amplification often exists within a larger network of cooperating oncogenic events rather than as an isolated driver. One particularly relevant example is PIKE-A/AGAP2, an AKT-enhancing GTPase encoded within the 12q13-15 region. PIKE-A amplification can accompany CDK4 amplification, and co-expression of PIKE-A and CDK4 accelerated gliomagenesis in a TP53/PTEN-deficient mouse model, providing direct evidence that co-amplified genes can cooperate functionally [26]. Under metabolic stress, PIKE-A can also interact directly with CDK4 in an AMPK-regulated manner, illustrating that the relationship between these proteins is context-dependent and may differ between nutrient-replete and energy-stressed tumor states [27].

Genomic alterations also influence GBM cellular state. In an interesting study by Neftel et al., single-cell analyses revealed that malignant GBM cells occupy multiple plastic transcriptional states and that genetic alterations help bias tumors toward particular states. For instance, CDK4 amplification was associated with a neural-progenitor-like program [23]. Consistent with this, CDK4/6 inhibition has demonstrated greater activity in subsets of proneural glioma stem-like cells with intact RB and elevated CDK6, while RB-deficient models tend to be resistant [24]. These observations are important because they shift interpretation away from a simple “CDK4-high equals sensitive” model. Pathway dependence is determined by the entire circuit, including CDKN2A/B, RB1, cyclin D, CDK4/6, E2F output, and upstream RTK/PI3K signaling, as well as the cellular state in which those alterations occur. Table 1 shows the comparative implications of CDK4/6-RB Pathway Alterations for CDK4/6 Inhibitor Response in GBM.

### 2.3. Noncanonical Functions and Regulatory Complexity of CDK4/6 in GBM

Although CDK4 and CDK6 are classically defined by their role in cell-cycle progression, accumulating evidence indicates that their contribution to GBM biology extends beyond proliferation alone. Their canonical function nevertheless provides an important framework for understanding these broader effects. Activation of the cyclin D-CDK4/6-RB-E2F axis sustains proliferative capacity, whereas pharmacologic CDK4/6 inhibition induces G1 arrest and suppresses intracranial xenograft growth in an RB-dependent manner [12]. Patient-derived xenograft (PDX) studies similarly demonstrate that the configuration of the p16-CDK4-RB axis influences therapeutic response, underscoring that dependence on CDK4/6 signaling is shaped by the molecular context of the tumor [11]. More recently, Mekonnen et al. confirmed using a CNS-optimized CDK4/6 inhibitor that this core pathway remains pharmacologically actionable when adequate target engagement is achieved [28]. Beyond this canonical cell-cycle function, however, CDK4/6 signaling appears to influence several additional features of GBM biology. Importantly, not all of these effects have been shown to occur independently of RB-mediated cell-cycle control. The strongest evidence for a truly RB-independent function comes from cytoplasmic cyclin D1-CDK4 signaling, which regulates focal-adhesion and invasive programs through RalA, Rac1, and paxillin in GBM [29,30]. In contrast, reported effects on stemness and cell-state plasticity, DNA-damage recovery, and CDK6 protein stability remain partly linked to canonical cell-cycle regulation or have not been shown conclusively to occur independently of RB signaling [24,31,32].

CDK4/6 signaling intersects with glioma stemness and phenotypic plasticity, as shown by Li et al. [24]. In proneural glioma stem-like cells, CDK4/6 inhibition preferentially suppresses proliferation but can induce a shift toward mesenchymal-associated programs, suggesting that cell-cycle blockade may alter tumor state rather than simply eliminate tumor cells [24]. More recent mechanistic work identified CDK4 as a regulator of a hybrid stem-like and invasive phenotype in GBM, linking CDK4 activity to paxillin-dependent cytoskeletal programs and stemness-associated behavior [33]. These findings complement earlier single-cell studies showing that CDK4 copy-number alterations influence neural-progenitor-like states [23] and support a broader role for CDK4 and potentially the CDK4/6 axis in shaping the balance between proliferation, stemness, and invasion. However, these studies do not fully separate changes in stemness or cell state from RB-dependent effects on proliferation and treatment selection, so this evidence is better viewed as supportive of a broader CDK4/6 role rather than definitive proof of an RB-independent mechanism [23,24].

A particularly important noncanonical function is regulation of invasion. As discussed above, cytoplasmic cyclin D1-CDK4 activity can promote GBM invasion independently of canonical nuclear RB signaling by activating focal-adhesion-associated effectors including RalA, Rac1, and paxillin [29]. Among the noncanonical functions discussed here, this represents the clearest GBM-specific evidence for an RB-independent role of CDK4 [29]. This provides a mechanistic explanation for why high cyclin D1-CDK4 activity may influence tumor spread even when changes in bulk proliferation are mild to modest. The observation is clinically relevant because diffuse infiltration into surrounding brain parenchyma is a defining feature of GBM and a principal reason that complete surgical eradication is difficult.

CDK4/6 signaling also plays an important role in DNA damage responses and radiation resistance. Palbociclib has been shown to impair DNA damage repair after ionizing radiation and delay regrowth of irradiated intracranial GBM xenografts [31]. Similarly, miR-124-mediated suppression of CDK4 radiosensitized glioma cells, further supporting a relationship between CDK4 activity and radiation response [34]. These effects should not be interpreted to mean that CDK4/6 directly blocks apoptosis in all contexts; rather, sustained CDK4/6-RB-E2F activity allows damaged cells to maintain or re-enter the proliferative program, while CDK4/6 inhibition can reinforce cell-cycle arrest and alter the capacity for post-radiation recovery. The distinction is important because CDK4/6 inhibitors are often predominantly cytostatic, and durable tumor control may require combination strategies that convert arrest into irreversible loss of clonogenic potential.

CDK6 is additionally regulated through post-translational mechanisms with consequences for GBM progression. Bellail et al. showed that SUMO1 conjugation stabilizes CDK6 by limiting ubiquitin-mediated degradation, maintaining CDK6 protein through the cell cycle and promoting G1/S progression, while suppression of this SUMOylation pathway reduced GBM growth and tumorigenicity [32]. Together with earlier observations involving p21, p27, proteasomal turnover, HSP90 chaperoning, and FAK/RTK signaling, these data emphasize that CDK4/6 abundance and activity are controlled at multiple levels and not only by gene copy number, but also by protein stability, localization, and upstream signaling [11,12].

Regulatory noncoding RNAs add another layer of control. Several miRNAs described in GBM converge on the CDK4/6 axis. miR-138 suppresses an EZH2-CDK4/6-RB-E2F1 signaling loop and reduces tumorigenicity, while miR-129 can directly target CDK4, CDK6, and MDM2 [35,36]. Rather than treating each miRNA as an independent pathway, these studies are best viewed collectively as evidence that the CDK4/6-RB checkpoint functions as a recurrent downstream node for diverse transcriptional and post-transcriptional regulatory programs.

Metabolic adaptation provides a further connection between cell-cycle signaling and the hostile GBM microenvironment. GBM cells must continually adjust to hypoxia, glucose deprivation, and fluctuating nutrient availability. The LKB1-AMPK axis coordinates a proliferation-versus-migration switch during metabolic stress, and AMPK is found to be required for bioenergetic adaptation in glioma stem-like cells [37,38]. More recent work confirms that AMPK supports adaptation of GBM cells to tumor-microenvironmental stressors such as hypoxia and nutrient limitation [39]. Within this framework, the AMPK-dependent regulation of PIKE-A and CDK4 provides a direct molecular bridge between energy sensing and the G1/S machinery [27]. These findings position CDK4 as a downstream component of a broader adaptive network linking cellular energetic state to proliferative control.

Finally, data has shown that CDK4-related genomic programs appear to interact with the immune microenvironment. Computational analysis associated CDK4 amplification with a more suppressive immune phenotype, including reduced lymphocyte/macrophage response signatures, and proposed co-amplified MARCH9 as one potential contributor [40]. Single-cell analysis subsequently showed that EGFR/CDK4 co-amplification is heterogeneous within tumors and is linked to patterns of immune infiltration, including macrophage-rich microenvironments [41]. These observations establish an association between CDK4-amplified tumor states and the immune microenvironment, although whether CDK4 activity itself causally remodels these niches remains unresolved. It is also important not to equate microglial activation or neuroinflammation with tumor-specific immune regulation. Guo et al. showed that laminarin-based platinum nanozymes can modulate inflammatory microglial responses after intracerebral hemorrhage through Dectin-1/Syk/NF-κB and oxidative-stress pathways [42]. However, this work was performed in a non-neoplastic injury model and does not establish a direct relationship with CDK4/6 inhibition or GBM treatment. Thus, while microglial state is clearly biologically important within the brain, evidence from inflammatory or injury models should be interpreted cautiously when discussing CDK4/6-associated immune effects in GBM.

**Figure 4:**
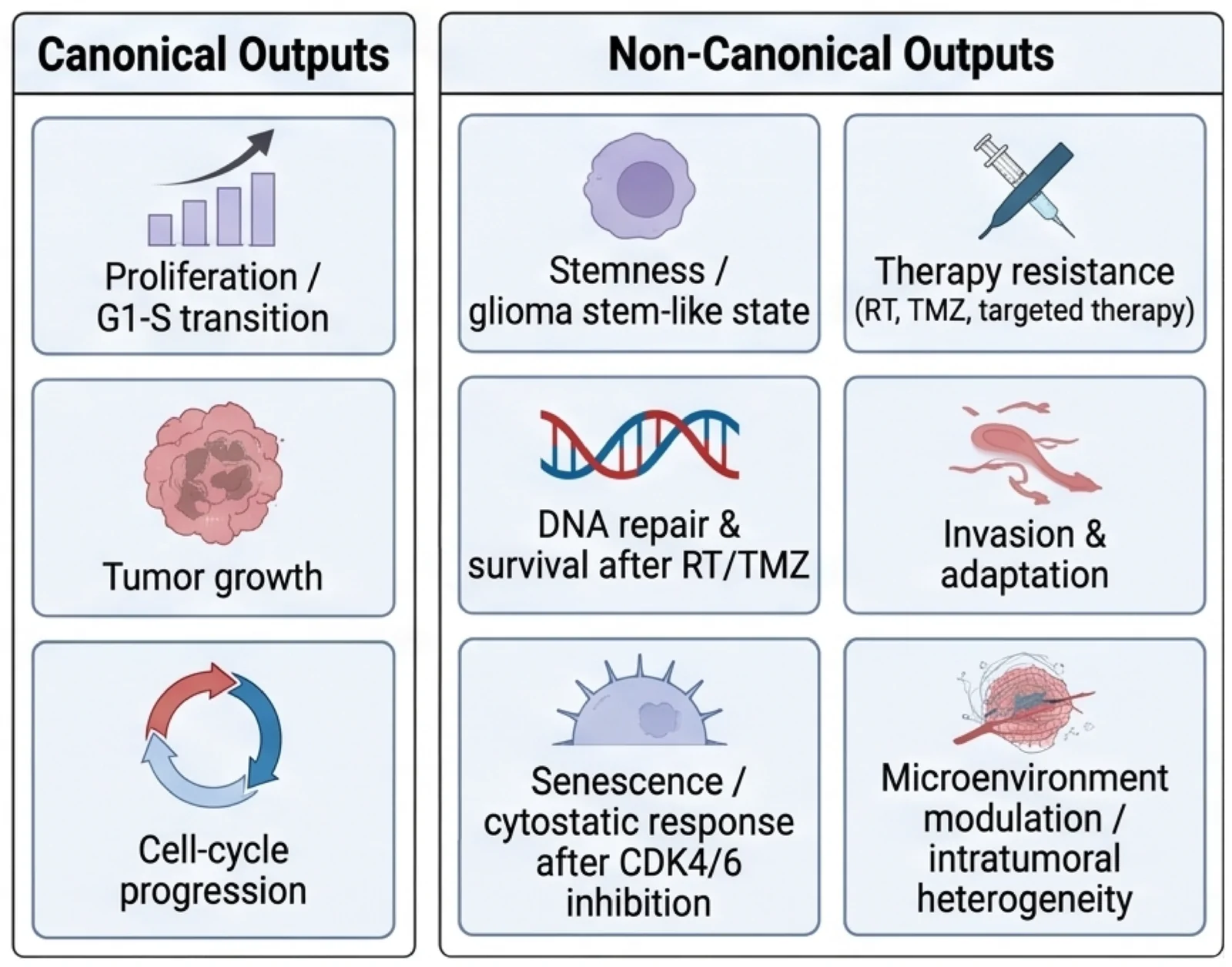
Canonical and non-canonical outputs of CDK4/6 signaling in glioblastoma. Canonical CDK4/6 activity promotes G1-S cell-cycle transition, continued cell-cycle progression, and tumor growth through regulation of the RB-E2F axis. Beyond these classical proliferative functions, CDK4/6 signaling is increasingly linked to non-canonical programs that influence glioblastoma biology and therapeutic response. These include maintenance of glioma stem-like states, resistance to radiotherapy, temozolomide, and targeted therapies, modulation of DNA repair and post-treatment survival, invasion and adaptive cellular programs, induction of senescence or cytostatic responses following CDK4/6 inhibition, and interactions with the tumor microenvironment and intratumoral heterogeneity. Together, these effects illustrate that CDK4/6 signaling extends beyond cell-cycle control and contributes to multiple processes relevant to glioblastoma progression and treatment resistance. Created in BioRender. Najjar, MK (2026). https://BioRender.com/qu7zyec.

In this context, it is important to distinguish the direct effects of CDK4/6 inhibition on tumor cells from its potential effects on the surrounding immune microenvironment. In GBM, the best established effects of CDK4/6 blockade remain tumor-cell intrinsic, as described previously in this review. In contrast, effects on the immune microenvironment are less well defined and may reflect changes in tumor-cell signaling, cytokine production, antigen presentation, or the composition and activation state of infiltrating immune cells rather than a uniform direct consequence of CDK4/6 inhibition itself. The broader glioma literature illustrates the importance of this immune context. Zeng et al. identified an inflammation-related lncRNA signature associated with glioma prognosis and immune infiltration and showed that LINC00346 expression correlated with type 2 macrophage-associated markers, including STAT3 and CD204 [43]. However, this study did not evaluate CDK4/6 inhibition and is better viewed as evidence that tumor-intrinsic transcriptional programs can shape the glioma immune milieu rather than as direct evidence of CDK4/6-mediated immune regulation. Similarly, Soltys et al. reviewed the current landscape of GBM immunotherapy, particularly CAR-T-cell approaches, and emphasized tumor heterogeneity, immunosuppressive myeloid and cytokine networks, T-cell dysfunction, and antigen variability as important barriers to durable immune responses [44]. These observations support consideration of the immune microenvironment when developing CDK4/6-based combinations, but they do not establish that CDK4/6 inhibition itself reverses immunosuppression or enhances cellular immunotherapy in GBM. Thus, although interactions between CDK4/6-associated tumor states and the immune microenvironment are increasingly recognized, whether CDK4/6 inhibition directly alters immune-cell recruitment or function in GBM remains unresolved. Taken together, these studies expand the biological significance of CDK4/6 dysregulation beyond its canonical role in G1/S progression. However, the strength of evidence varies across these proposed functions. GBM-specific mechanistic studies most clearly support an RB-independent role for cytoplasmic CDK4 in invasion and focal-adhesion signaling [29], while evidence linking CDK4/6 to stem-like states, phenotypic plasticity, and recovery after genotoxic stress is substantial but remains partly intertwined with canonical cell-cycle control [23,24]. By contrast, links to metabolic adaptation and the immune microenvironment are more exploratory and, in several cases, are based on associative findings or mechanistic extrapolation rather than direct demonstration of an RB-independent CDK4/6 function. These effects are highly context-dependent, are not necessarily shared equally by CDK4 and CDK6, and in some cases remain associative rather than directly mechanistic. This biological complexity may help explain why CDK4/6 remains an attractive therapeutic target despite the limited efficacy of monotherapy and further supports molecularly selected, rational combination strategies in GBM. Figure 4 summarizes the canonical and non-canonical biological outputs associated with the CDK4/6 pathway.

## 3. Pharmacology and CNS Delivery of CDK4/6 Inhibitors in Glioblastoma

Selective CDK4/6 inhibitors were developed to suppress cyclin D-CDK4/6 signaling by binding the ATP-binding pocket of CDK4 and CDK6, reducing RB phosphorylation and maintaining E2F-dependent transcription in a repressed state [9,16]. Although palbociclib, ribociclib, and abemaciclib share this canonical mechanism, interestingly, they are not pharmacologically interchangeable. They differ in relative potency for CDK4 versus CDK6, kinase selectivity, dosing schedule, systemic half-life, active metabolites, and, particularly important for GBM, their ability to reach pharmacologically active concentrations within the CNS [45,46,47]. While palbociclib is relatively balanced in its inhibition of CDK4 and CDK6, ribociclib and abemaciclib demonstrate a modest preference for CDK4 in cellular systems and abemaciclib also has broader kinase activity at higher concentrations than the other two agents [46,47]. Their clinical dosing reflects additional pharmacokinetic (PK) differences: palbociclib and ribociclib have terminal/effective half-lives on the order of approximately one day and are generally administered once daily, whereas abemaciclib has a shorter effective half-life and is administered continuously twice daily in a clinical setting [45].

For GBM, however, systemic PK alone is insufficient. The BBB remains relatively intact within infiltrative and non-enhancing regions of tumor, while the BTB within contrast-enhancing tumor is disrupted to a variable and spatially heterogeneous degree [48]. Importantly, BBB penetration and BTB penetration should not be considered equivalent. In contrast-enhancing regions of the tumor, disruption of the BTB may allow greater drug exposure, whereas infiltrative and non-enhancing tumor margins often retain a more intact BBB and may therefore be exposed to substantially lower free drug concentrations [48]. Thus, detecting a drug within the enhancing portion of the tumor does not necessarily mean that pharmacologically active concentrations are achieved throughout the infiltrative compartment, a distinction that is particularly important when comparing the apparent CNS penetration of different CDK4/6 inhibitors. Studies have shown that active efflux by P-glycoprotein (P-gp/ABCB1) and breast cancer resistance protein (BCRP/ABCG2) further limits the intracranial distribution of several CDK4/6 inhibitors [49,50,51]. Emerging delivery strategies may also provide approaches for overcoming regional BBB/BTB limitations. For instance, Wang et al. developed cRGD-modified liposomes designed to enhance BBB/BTB penetration and tumor targeting in an orthotopic GBM model [52]. Although this work supports the broader feasibility of engineered carriers for improving drug delivery to treat GBM, the platform was not used to deliver a CDK4/6 inhibitor and therefore should not be interpreted as direct evidence that liposomal formulation improves CDK4/6 inhibitor exposure in GBM. These considerations make direct measurement of tumor PK and pharmacodynamics (PD) particularly important over plasma or cerebrospinal fluid (CSF) concentrations alone. Phase 0 and window-of-opportunity studies have become central to the development of targeted agents for GBM because they can determine whether a compound actually penetrates enhancing and non-enhancing tumor at an adequate unbound concentration and suppresses its intended pathway before large efficacy trials are undertaken [53]. Table 2 summarizes the relative characteristics of the three clinically available and selected emerging CDK4/6 inhibitors.

### 3.1. Palbociclib

Palbociclib was the first selective CDK4/6 inhibitor to establish the therapeutic feasibility of this drug class and was also the first to be studied extensively in GBM. Early preclinical work provided a strong biological rationale when Michaud et al. demonstrated that palbociclib produced G1 arrest in RB-proficient GBM cell lines, while RB-deficient lines were resistant, establishing functional RB as a prerequisite for drug activity [12]. Subsequent studies in patient-derived glioma stem cell models revealed additional context dependence, with preferential sensitivity of proneural GBM models and resistance associated with RB1 loss, while prolonged CDK4/6 inhibition produced a largely reversible quiescent state rather than a terminally senescent state [24,54].

The principal pharmacologic weakness of palbociclib in GBM is optimal CNS delivery. Although early orthotopic studies demonstrated measurable brain and tumor exposure, later transporter-focused studies clarified that palbociclib is efficiently exported by both ABCB1 and ABCG2 at the BBB [49,55]. Parrish et al. demonstrated a striking increase in brain concentrations of palbociclib in mice lacking these efflux transporters and showed that a dose effective against a subcutaneous GBM xenograft was ineffective against the same model implanted intracranially because brain exposure was insufficient [55]. Thus, the limitation is best considered PK resistance rather than intrinsic tumor-cell resistance, and thus, the drug can inhibit susceptible GBM cells when adequate concentrations are achieved.

From a pharmacologic perspective, its major limitations remain strong BBB efflux, heterogeneous tumor exposure, and a predominantly cytostatic effect. However, the failure of palbociclib in recurrent GBM cannot be attributed to CNS delivery alone, because clinically relevant tumor concentrations were achieved in several patients without consistent pharmacodynamic or clinical benefit. These findings argue against unselected monotherapy and favor biomarker-selected or combination strategies. Clinical efficacy outcomes for palbociclib are discussed in Section 6.

### 3.2. Ribociclib

Ribociclib is also a highly selective CDK4/6 inhibitor, with somewhat greater activity against CDK4 than CDK6 based on cellular assays [47]. Systemically, it has a relatively long half-life: the recommended 600-mg dose in its first-in-human study was associated with a mean effective half-life of approximately 32.6 h [56]. Like palbociclib, its CNS distribution is constrained by active transport. Preclinical PK studies demonstrated that ABCB1 can markedly restrict ribociclib brain concentrations, although its passive permeability permits measurable CNS penetration and the magnitude of this limitation differs from that of palbociclib [51]. These observations highlight why classifying a drug simply as “BBB penetrant” or “non-penetrant” can be misleading. Thus, the clinically relevant question should be whether free drug concentrations within the tumor exceed the concentration required for sustained target inhibition.

Ribociclib has provided one of the clearest demonstrations of phase 0 pharmacology in GBM. In recurrent GBM, presurgical ribociclib achieved pharmacologically relevant concentrations in both enhancing and non-enhancing tumor, with decreased RB phosphorylation and tumor-cell proliferation [14]. This work establishes that ribociclib can reach tumor and engage its target.

More recent phase 0/1 work has extended this concept by combining ribociclib with mTOR inhibition and directly profiling treated tumor tissue. In recurrent high-grade glioma, including IDH-wildtype tumors, unbound ribociclib concentrations in non-enhancing tissue exceeded the biochemical CDK4/6 inhibitory range and were associated with reduced Ki-67 and shifts away from highly cycling malignant cell states. In contrast, everolimus exposure in tumor was very low in this study [57]. Although this study was not restricted exclusively to GBM, it reinforces the translational value of measuring each component of a combination independently within tumor and supports the claim that ribociclib can reach tumor and engage its target. For ribociclib, the major limitation therefore appears less related to inadequate tumor penetration than to the inability of CDK4/6 inhibition alone to prevent adaptive and compensatory signaling pathways from restoring tumor proliferation.

**Table 2:** Comparative Pharmacology of CDK4/6 Inhibitors Relevant to GBM.

Agent	CDK4/CDK6 Activity	Dosing/Stage	CNS/BBB Profile	Efflux Liability	GBM-Relevant Evidence	Development Status	Refs
Palbociclib	balanced CDK4/CDK6 activity	Oral daily; ~1 day half-life	Low heterogenous tumor exposure	ABCB1/ABCG2 efflux	RB-dependent preclinical arrest; recurrent GBM monotherapy negative despite measurable tumor exposure	Approved in breast cancer; GBM trial completed	[11,12,45,49,55]
Ribociclib	modest CDK4 preference	Oral daily; ~32 h half-life	Phase 0: Measurable enhancing and non-enhancing tumor exposure	ABCB1 efflux	Phase 0 pRB/Ki-67 suppression, but short PFS: target engagement ≠ durable benefit	Approved in breast cancer; GBM phase 0 completed	[14,45,47,50,58]
Abemaciclib	Selective CDK4 preference; broader activity at higher doses	Oral BID; continuous dosing;	Best relative CNS exposure of approved agents; still heterogenous	ABCB1/ABCG2 efflux	Preclinical brain exposure and TMZ synergy; limited clinical benefit despite CNS penetration	Approved in breast cancer; GBM studies ongoing/completed	[15,45,46,50,59,60]
Brain-penetrant CDK4/6-IN series	Selective; designed to spare CDK1/2	Preclinical	Optimized brain exposure	Reduced efflux by design	Activity in RB-wildtype GBM-relevant models; no clinical efficacy data	Preclinical only	[61]
SPH3643	Potent CDK4/6 inhibition	Preclinical	High intracerebral exposure	Favorable preclinical profile	Orthotopic tumor suppression; clinical activity unknown	Preclinical only	[62]
GLR2007	Investigational CDK4/6 inhibitor	Preclinical/investigational	Preclinical brain levels exceed plasma	CNS-optimized profile	Orthotopic IDH-wildtype GBM activity; human efficacy unknown	Investigational	[63,64]
AU3-14	CNS-optimized CDK4/6 inhibitor	Preclinical; early clinical development	Favorable unbound brain exposure	Low predicted P-gp liability	Orthotopic efficacy and TMZ enhancement; mature GBM efficacy not available	Preclinical/early clinical	[28]

### 3.3. Abemaciclib

Among the three clinically available selective CDK4/6 inhibitors, abemaciclib has the most favorable overall CNS pharmacologic profile. It has a greater proclivity for CDK4, has greater passive membrane permeability, and although it remains susceptible to ABCB1/ABCG2-mediated efflux, it achieves substantially greater concentrations in the brain than palbociclib in preclinical models [15,46,50]. Raub et al. directly compared abemaciclib and palbociclib in intracranial glioma models and found higher brain concentrations with abemaciclib [15]. Later studies confirmed that ABCB1 and ABCG2 still restrict abemaciclib and its active metabolites, emphasizing that its superior CNS profile is relative rather than evidence of unrestricted BBB passage [50].

Early clinical pharmacology also supports CNS penetration of abemaciclib. In first-in-human studies, 17 patients with GBM were included among 225 participants, and abemaciclib was detectable in CSF [59]. These observations motivated disease-specific studies, whose efficacy results are discussed in Section 6. Pharmacologically, abemaciclib’s principal advantage over palbociclib and ribociclib is therefore improved CNS exposure rather than demonstrated clinical superiority in GBM.

The distinction between CSF and tumor exposure is especially relevant to abemaciclib. CSF sampling can demonstrate that a drug enters the CNS, but CSF concentrations do not necessarily correspond to the unbound extracellular concentration within enhancing tumor, infiltrative non-enhancing tumor, or individual cellular niches. Current neuropharmacology studies are therefore moving toward direct intratumoral measurements. A 2023 clinical protocol described perioperative abemaciclib administration followed by intratumoral microdialysis to measure extracellular drug concentrations over time, illustrating a strategy that may more accurately define pharmacologically active exposure than plasma or CSF concentrations alone [65]. Although that protocol encompasses recurrent high-grade/diffuse midline gliomas rather than GBM exclusively, the methodology is directly relevant to the central drug-delivery problem in GBM. Taken together, the available evidence makes abemaciclib the most pharmacologically attractive of the approved agents for further GBM investigation, particularly in biomarker-selected patients and combination regimens. Its superior CNS exposure, however, has not yet translated into clear clinical superiority over the other approved CDK4/6 inhibitors.

When considered together, however, the relative advantages of the three FDA-approved CDK4/6 inhibitors are more nuanced than their pharmacologic profiles alone suggest. Palbociclib has the least favorable CNS profile, with substantial ABCB1/ABCG2-mediated efflux in preclinical models. However, its negative phase II study demonstrated adequate tumor concentrations in several patients without consistent PD or clinical benefit, indicating that drug delivery alone does not explain its limited activity in GBM [13]. Ribociclib has the strongest direct clinical evidence of target engagement. Phase 0 studies demonstrated pharmacologically relevant unbound concentrations in enhancing and non-enhancing tumors together with reduced RB phosphorylation and proliferation, yet monotherapy produced only limited disease control [14]. As discussed above, abemaciclib has the most favorable overall CNS pharmacology among the approved agents and has demonstrated drug exposure and RB-pathway suppression in human GBM tissue. Unfortunately, clinical activity has also remained limited, including in molecularly selected recurrent GBM [66]. Thus, improved CNS exposure and target engagement appear necessary but insufficient for efficacy. Among the approved agents, abemaciclib remains pharmacologically attractive for GBM-directed combinations, while ribociclib provides particularly strong proof that successful tumor penetration and pathway inhibition do not, by themselves, translate into durable clinical benefit.

### 3.4. Emerging Brain-Penetrant CDK4/6 Inhibitors

The limitations of the approved agents have stimulated development of CDK4/6 inhibitors designed from the outset for improved CNS exposure. An early medicinal-chemistry program produced “compound 11” (later known as GLR2007), a potent CDK4/6 inhibitor optimized for BBB penetration that achieved favorable brain concentrations and prolonged survival in intracranial GBM models without major body-weight loss [63]. Another study described a brain-penetrant CDK4/6 inhibitor generated by reducing molecular weight and polar surface area, tuning basicity, and minimizing transporter-mediated efflux while preserving selectivity against CDK1/2 [61]. These studies established that CNS pharmacology can be engineered rather than accepted as a fixed property of the class, opening up a door for another sphere of research.

SPH3643 subsequently demonstrated potent inhibition of CDK4/6, strong BBB permeability, persistent intracerebral exposure, and marked suppression of orthotopic U87-MG tumor growth in mice [62]. Yin et al. extended this approach in IDH-wildtype GBM models using GLR2007 [64]. In this preclinical study, the authors reported brain drug levels several-fold higher than plasma and substantial growth inhibition in orthotopic GBM models [64]. Neither of the two aforementioned compounds has yet established clinical efficacy in GBM, but both illustrate the increasing emphasis on integrating potency, selectivity, intracranial concentrations, and durability of target inhibition during lead optimization.

The most recent example is AU3-14, reported in 2025 as a brain-penetrant CDK4/6 inhibitor specifically developed for GBM. AU3-14 showed high permeability, low predicted P-gp liability, favorable unbound brain-to-plasma partitioning, and intracerebral exposure reported to exceed that of abemaciclib in preclinical comparisons [28]. This drug inhibited tumor growth in orthotopic GBM models while maintaining a favorable preclinical safety profile in rats and nonhuman primates [28]. These data are particularly relevant because they move beyond total brain concentration toward unbound exposure, which is a more meaningful measure of the pharmacologically available drug fraction. AU3-14 remains preclinical/investigational, but it exemplifies the design characteristics that future GBM-directed CDK4/6 inhibitors will likely require.

## 4. Mechanisms of Resistance to CDK4/6 Inhibition in Glioblastoma

Resistance to CDK4/6 inhibition in GBM is a multifactorial process rather than a single molecular event. The available GBM-specific literature remains smaller than the resistance literature in breast cancer, but several recurring themes have emerged: loss of dependence on the canonical RB checkpoint, compensatory activation of growth-factor and survival pathways, transcriptional and cell-state plasticity, persistence of glioma stem cells, reversible cytostasis, and inadequate drug exposure within the brain. These mechanisms are not mutually exclusive and may coexist within the same tumor because GBM contains genetically and phenotypically distinct cellular populations. Importantly, some resistance mechanisms established in other cancers, particularly cyclin E-CDK2 bypass and E2F reactivation, are biologically plausible in GBM but have not yet been demonstrated as rigorously in GBM models. The discussion below therefore distinguishes mechanisms supported directly by GBM data from mechanisms that remain candidate pathways requiring validation [67,68]. Importantly, cell-cycle escape and parallel survival signaling should not be viewed as separate processes. Even when CDK4/6 blockade constrains RB-E2F-driven cell-cycle progression, persistent or treatment-induced activation of PI3K-AKT-mTOR, MAPK/ERK, or receptor tyrosine kinase signaling may preserve metabolism, survival, and mitogenic competence, allowing viable tumor cells to persist and later recover proliferative capacity. The strength of evidence for these mechanisms differs, however, and the sections below distinguish pathways directly shown to mediate resistance during CDK4/6 inhibition from signaling networks that remain biologically plausible but incompletely validated in this specific context.

### 4.1. Intrinsic Genomic Resistance and Cell-Cycle Bypass

The most direct form of resistance is loss of dependence on the CDK4/6-RB axis. CDK4/6 inhibitors require functional RB to translate kinase inhibition into durable suppression of E2F-dependent G1/S progression. In GBM in vitro models, RB-deficient cells are resistant to palbociclib, whereas RB-proficient models can undergo profound G1 arrest [12]. The p16INK4A-CDK4/6-RB context further modifies sensitivity. In a panel of patient-derived GBM xenografts, Cen et al. found that tumors with CDKN2A/B deletion, non-amplified CDK4, and wild-type RB were particularly susceptible to palbociclib, while models retaining p16INK4A together with CDK4 amplification or an RB mutation were resistant [11]. Thus, CDKN2A loss should not automatically be interpreted as a resistance marker because in an RB-intact tumor it may instead indicate stronger dependence on CDK4/6 signaling. Similarly, amplification of CDK4 does not guarantee drug sensitivity and may identify a cell-cycle configuration capable of escaping pharmacologic inhibition.

A second route is restoration of S-phase entry through CDK4/6-independent machinery. In breast cancer, CCNE1 amplification, increased cyclin E-CDK2 activity, RB1 loss, and reactivation of E2F programs are established mechanisms of acquired CDK4/6 inhibitor resistance [67,69]. Direct evidence for cyclin E-CDK2-driven resistance in GBM remains limited, so this pathway should be considered a candidate rather than a confirmed GBM mechanism. Nevertheless, the biology is highly relevant because E2F activation is the downstream event that CDK4/6 inhibition is designed to suppress. Any alteration that preserves RB phosphorylation or activates CDK2/E2F independently of CDK4/6 could restore proliferation despite adequate drug exposure. This provides a rationale for profiling RB1, CDKN2A/B, CDK4, CDK6, CCNE1, CDK2, and E2F activity together rather than using a single genomic alteration as a biomarker.

### 4.2. Adaptive Signaling and Compensatory Pathway Activation

GBM cells can also adapt to CDK4/6 inhibition by activating parallel survival pathways. One of the strongest and classic GBM-specific examples involves PI3K-AKT-mTOR signaling. Olmez et al. showed that while palbociclib initially suppresses mTOR signaling, it produces rebound mTOR activation, thereby identifying a compensatory response to CDK4/6 blockade [70]. This adaptive signaling is relevant because CDK4/6 inhibition is predominantly cytostatic and a cell that has slowed or stopped G1/S progression can remain viable if parallel pathways continue to support protein synthesis, metabolism, and survival. In the same study, co-treatment with everolimus blocked the rebound response and increased antitumor activity, linking survival-pathway compensation directly to the durability of cell-cycle blockade [70]. The interaction is bidirectional such that mTOR inhibition can increase ERK signaling, while CDK4/6 inhibition can suppress that ERK rebound, illustrating the extensive feedback architecture connecting cell-cycle control with mitogenic signaling. Phase 0 studies of ribociclib have similarly emphasized that achieving RB pathway inhibition in GBM does not necessarily produce durable tumor control, reinforcing the concept that target engagement may be followed by compensatory survival signaling [14].

Additional evidence implicates PTEN, AKT, and ERK as determinants of drug sensitivity [17,71]. Restoration or expression of PTEN increased GBM sensitivity to palbociclib and was associated with suppression of AKT and ERK signaling, whereas PTEN-deficient signaling reduced the antiproliferative effect [71]. Recent patient-derived models further support PI3K activity as an escape pathway during CDK4/6 inhibition in a study by Zhang et al. [17]. Together, these studies support PI3K-AKT-ERK activity as a modifier of the durability and magnitude of CDK4/6 response, but they do not imply that every alteration in these pathways is itself a validated resistance mechanism. The strongest interpretation is that persistent or rebound survival signaling can allow tumor cells to tolerate CDK4/6-mediated arrest and provide an alternative route to proliferative recovery.

RTK re-circuitry represents another well-defined adaptive mechanism. Olmez and colleagues showed that abemaciclib treatment activated c-MET and TrkA/TrkB in glioma-initiating cells. Mechanistically, CDK4/6 inhibition induced NF-κB-dependent upregulation of HGF, BDNF, and NGF, which stimulated the corresponding c-MET and Trk pathways. Dual c-MET/Trk blockade restored sensitivity, supporting these receptors as adaptive resistance nodes [72]. This represents direct evidence of treatment-induced bypass because the alternative signaling program emerged during CDK4/6 inhibition and pharmacologic blockade of that program restored response.

Several other signaling networks are implicated in glioma biology, although their direct roles in mediating resistance to CDK4/6 inhibition remain unestablished. Liu et al. demonstrated that miR-383-5p suppresses glioma-cell proliferation and invasion through inhibition of a VEGFA-AKT-mTOR signaling axis, supporting the broader importance of AKT/mTOR signaling in glioma growth and survival [73]. However, that study did not evaluate a CDK4/6 inhibitor and therefore should be interpreted as evidence for an alternative survival network rather than as direct evidence of CDK4/6 resistance. Similarly, in a more recent study, Liu et al. showed that miR-124-3p suppresses glioma progression through a STAT3/NAMPT-associated AKT/ERK signaling network [74]. This work further illustrates the ability of glioma cells to use parallel signaling circuits that could plausibly sustain survival or proliferation when the canonical CDK4/6-RB axis is restrained. Similar to the previous study, CDK4/6 blockade was not evaluated and a role in resistance remains unproven.

Thus, the available data support a hierarchy of evidence. Rebound mTOR signaling and treatment-induced c-MET/Trk activation have been demonstrated directly in GBM models during CDK4/6 inhibition and represent established adaptive resistance mechanisms [70,72]. PTEN/AKT/ERK signaling and PI3K co-activation have strong mechanistic evidence as modulators of CDK4/6 response and potential escape pathways [17,71]. By contrast, other AKT, ERK, STAT3, and metabolic signaling programs identified in glioma should remain classified as plausible bypass mechanisms until they are shown experimentally to preserve proliferation or viability during CDK4/6 blockade [73,74]. This distinction is important because it avoids equating general glioma survival signaling with mechanisms specifically demonstrated to cause CDK4/6 inhibitor resistance.

### 4.3. Transcriptional Reprogramming, Stemness, and tGLI1-Mediated Resistance

Resistance may also arise from transcriptional programs that preserve stemness and survival without requiring a new driver mutation. A particularly relevant emerging mechanism is the truncated glioma-associated oncogene transcription factor (tGLI1). In a recent study from our group, we identified tGLI1 as a mediator of GBM resistance to CDK4/6 inhibitors. tGLI1-expressing GBM and glioma stem-like cells were less responsive to CDK4/6 blockade, whereas pharmacologic targeting of tGLI1 with ketoconazole increased sensitivity to CDK4/6 inhibition, reduced stemness-associated phenotypes, and enhanced apoptosis in in vitro and in vivo models [75]. This study directly links a GBM-enriched transcriptional program with CDK4/6 inhibitor response.

Epigenetic regulation may contribute to this transcriptional flexibility. In glioma models, inhibition of the HOTAIR-EZH2 axis with AC1Q3QWB promoted beta-catenin degradation and enhanced the antitumor activity of palbociclib in vitro and in vivo [76]. This does not by itself establish HOTAIR/EZH2 signaling as a universal acquired resistance mechanism, but it supports the broader concept that epigenetic and Wnt-associated transcriptional states can modulate dependence on CDK4/6 and create drug-tolerant populations.

### 4.4. Cell-State Plasticity and Intratumoral Heterogeneity

Cell-state plasticity is especially important in GBM because malignant cells can transition among neural-progenitor-like, oligodendrocyte-progenitor-like, astrocyte-like, and mesenchymal-like programs in response to genetic and microenvironmental pressures [23]. This dynamic organization means that a CDK4/6 inhibitor may preferentially suppress one cell state while allowing another to persist or expand. In patient-derived glioma stem-cell models, proneural cells were more sensitive to palbociclib, but treatment increased mesenchymal-associated markers and decreased proneural markers, consistent with therapy-induced or therapy-selected proneural-to-mesenchymal transition [24]. On the contrary, targeting ALDH1A3, a regulator of the mesenchymal glioma stem-cell state, improved the activity of palbociclib in these models [24,77].

These findings provide a mechanistic bridge between intratumoral heterogeneity and acquired drug resistance. Rather than acquiring a single resistance mutation, GBM may evade therapy by changing the relative abundance of pre-existing cellular states or by reprogramming surviving cells into a more resistant phenotype. Longitudinal studies have shown that GBM transcriptional subtypes can change at recurrence and that these changes are accompanied by alterations in the tumor microenvironment [78,79,80,81,82,83]. Consequently, a pretreatment biopsy alone may incompletely predict CDK4/6 dependence throughout therapy, and serial molecular assessment may ultimately be required to understand evolving resistance.

### 4.5. Reversible Senescence-like Cell Arrest and Drug-Tolerant Persistence

A conceptually distinct limitation is that CDK4/6 inhibition may be predominantly cytostatic rather than cytotoxic [24,84]. In patient-derived glioma stem cell-enriched cultures, palbociclib produced a senescence-like phenotype characterized by cell-cycle arrest, flattened morphology, beta-galactosidase activity, and induction of senescence-associated markers. However, these cells retained viability and resumed proliferation after drug withdrawal, indicating a reversible quiescent state rather than true, irreversible senescence [54]. This distinction is important because decreased proliferation during treatment does not necessarily indicate durable antitumor activity. True senescence implies sustained loss of proliferative capacity despite drug removal, whereas apoptosis reflects tumor-cell death. In contrast, reversible cytostasis leaves a viable population capable of re-entering the cell cycle and potentially contributing to recurrence. This form of drug-tolerant persistence may explain why robust short-term suppression of proliferation does not necessarily translate into durable tumor control. It also suggests that continuous exposure, strategies that convert cytostasis into apoptosis, or combinations with agents that eliminate senescent-like persister cells may be necessary for durable responses. Thus, preclinical studies should assess recovery after drug withdrawal, clonogenic regrowth, and cell death in addition to short-term measures of proliferation or tumor-volume reduction. In this context, sustained loss of clonogenic capacity may be a more meaningful indicator of durable efficacy than transient G1 arrest alone.

### 4.6. Pharmacologic Resistance and Inadequate Intratumoral Exposure

Finally, resistance may occur even when tumor cells remain biologically sensitive if sufficient drug never reaches infiltrative disease. Palbociclib is a substrate of both P-glycoprotein (ABCB1) and BCRP (ABCG2), and genetic loss of these transporters in mice markedly increases brain exposure [49]. An orthotopic study demonstrated that palbociclib could inhibit the same GBM model when implanted subcutaneously but was ineffective intracranially because BBB efflux limited drug delivery [55]. This is pharmacologic rather than tumor-cell resistance and should be interpreted separately from genomic or adaptive mechanisms. The key takeaway point here is that inadequate free intratumoral exposure can coexist with and may amplify biologic resistance.

**Figure 5:**
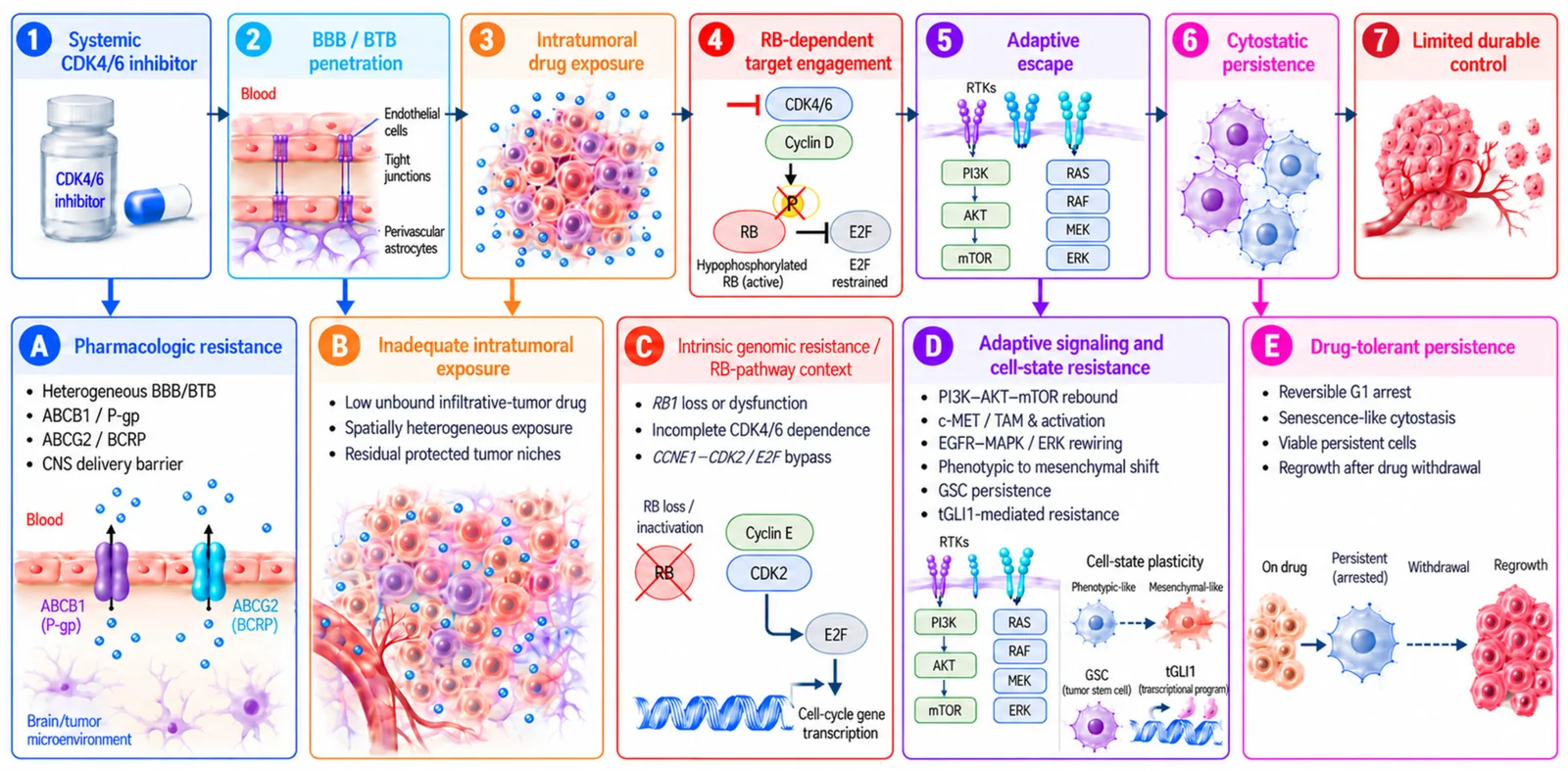
Mechanisms of resistance to CDK4/6 inhibition in glioblastoma. Therapeutic resistance to CDK4/6 inhibition in glioblastoma arises through multiple pharmacologic, genomic, signaling, and cell-state mechanisms. Systemically administered CDK4/6 inhibitors must first overcome heterogeneous blood–brain barrier and blood–tumor barrier permeability and active efflux through transporters such as ABCB1/P-glycoprotein and ABCG2/BCRP, which can limit CNS delivery and produce spatially heterogeneous intratumoral drug exposure. Even when adequate tumor concentrations are achieved, effective target engagement depends on an intact RB-E2F axis; RB1 loss or dysfunction, incomplete dependence on CDK4/6, and bypass through Cyclin E-CDK2-E2F signaling can confer intrinsic resistance. Additional adaptive mechanisms include rebound activation of PI3K-AKT-mTOR and RAS-RAF-MEK-ERK signaling, receptor tyrosine kinase rewiring, mesenchymal cell-state transitions, persistence of glioma stem cells, and tGLI1-associated transcriptional programs. CDK4/6 inhibition may also induce a predominantly cytostatic state characterized by reversible G1 arrest, senescence-like persistence, and survival of drug-tolerant persister cells, permitting tumor regrowth after treatment withdrawal. All in all, these mechanisms contribute to the limited durability of CDK4/6-directed therapy in glioblastoma and provide a rationale for combination strategies designed to improve drug delivery, prevent pathway bypass, and eliminate persistent tumor-cell states.

Taken all these together, resistance to CDK4/6 inhibition in GBM reflects at least three interacting problems: (1) an incomplete dependence on the RB checkpoint, (2) rapid adaptive re-circuitry of oncogenic signaling and cellular states, and (3) persistence of viable tumor cells during cytostatic arrest. These problems argue against CDK4/6 monotherapy and support a strategy combining molecular selection, confirmation of tumor drug exposure and target engagement, and rational co-targeting of the dominant escape pathway. Figure 5 summarizes the mechanisms of resistance to CDK4/6 inhibition in GBM.

## 5. Preclinical Therapeutic Strategies

Given the limited activity of single-agent CDK4/6 inhibition in GBM, the greatest therapeutic value of this drug class may lie in combination therapies. CDK4/6 inhibitors primarily impose a cytostatic G1 arrest, whereas GBM cells retain multiple mechanisms for DNA repair, survival signaling, cell-state adaptation, and recovery after drug withdrawal. Combination strategies are therefore most compelling when the partner therapy either converts cell-cycle arrest into irreversible cell death or blocks a compensatory pathway activated by CDK4/6 inhibition. Preclinical studies support several such approaches, particularly combinations with radiotherapy, TMZ, and RTK/PI3K/AKT/mTOR pathway inhibitors.

The strength of evidence is not equivalent across these combinations. RT has the clearest evidence for schedule-dependent interaction with CDK4/6 inhibition, with both clonogenic and intracranial survival studies showing that concurrent treatment or CDK4/6 inhibition after irradiation is more effective than administering the inhibitor before radiation [31,85]. In contrast, the evidence for TMZ is more limited. Intracranial GBM xenograft studies with abemaciclib and newer brain-penetrant inhibitors have shown improved survival or delayed resistance, but these data do not consistently establish formal pharmacologic synergy, and the optimal sequence and duration remain uncertain [15]. On the contrary, combinations targeting RTK/PI3K/mTOR signaling have some of the strongest mechanistic evidence for genuine adaptive resistance, because CDK4/6 inhibition itself can induce compensatory signaling that is suppressed by the partner drug. Palbociclib plus everolimus and abemaciclib plus c-MET/Trk inhibition, for example, produced synergistic apoptosis and improved antitumor activity in GBM models rather than simply additive growth inhibition [70,72]. Thus, combinations should be evaluated not only by whether they outperform either monotherapy, but also by whether the interaction is mechanistically synergistic, schedule-dependent, and durable after treatment withdrawal.

Interpretation of these preclinical studies also depends heavily on the experimental models used. Much of the earlier CDK4/6 work relied on established GBM cell lines and conventional xenografts, which are useful for defining drug mechanism and pathway dependence but do not necessarily fully capture the molecular diversity, infiltrative behavior, or treatment history of human GBM. More recent studies using patient-derived glioma stem cells have shown that CDK4/6 responses can vary across cellular states and that treatment may leave behind viable, drug-tolerant populations [54]. This is particularly relevant in GBM, where single-cell studies have demonstrated extensive intratumoral heterogeneity and coexistence of multiple malignant cell states within the same tumor [23,86]. Patient-derived organoids and orthotopic models improve on established cell lines, but they still only partially reproduce the spatial heterogeneity and therapy-driven evolution seen in recurrent GBM. Thus, findings based mainly on established cell lines should be viewed as more hypothesis-generating, whereas results reproduced across patient-derived and orthotopic models provide stronger, although still preclinical, support for translation.

The translational value of these combinations also depends on whether both agents can achieve the exposures and concentrations required for activity in patient tumors. This remains uncertain for several preclinical studies, particularly those that rely on micromolar drug concentrations in vitro or animal dosing schedules that are difficult to reproduce clinically. Palbociclib-based combinations are one example, as commonly used preclinical doses can generate systemic exposures well above those typically achieved in patients, while BBB efflux further limits delivery to the brain [49]. A similar issue is seen with mTOR-directed combinations. Although CDK4/6 plus mTOR inhibition has shown strong activity in GBM models, a phase 0/1 study found pharmacologically relevant ribociclib concentrations in non-enhancing tumor while unbound everolimus remained below the limit of detection across the doses tested [57]. Clinical testing of abemaciclib with the ERK1/2 inhibitor LY3214996 has raised the same concern, which is that only a minority of patients achieved prespecified intratumoral PK thresholds for both agents, and ERK pathway suppression was inadequate [87]. These findings suggest that some combinations may fail in the clinic not because the underlying biology is incorrect, but because effective dual-pathway inhibition cannot be achieved throughout the tumor at tolerable systemic doses.

### 5.1. CDK4/6 Inhibition Combined with Radiotherapy

RT is one of the strongest preclinical partners for CDK4/6 inhibition. In RB-proficient intracranial GBM models, palbociclib suppressed tumor growth and prolonged survival, providing the foundation for combination studies for this drug class [12]. Hashizume et al. subsequently demonstrated that palbociclib enhanced the effect of ionizing radiation in RB-proficient GBM xenografts by delaying repair of radiation-induced DNA double-strand breaks and increasing apoptosis [31]. Importantly, treatment sequence mattered: concurrent palbociclib with radiation, or palbociclib administered after radiation, was more effective than giving palbociclib before irradiation [31]. This is biologically plausible because premature G1 arrest can reduce the fraction of cells traversing radiation-sensitive phases of the cell cycle, whereas post-radiation CDK4/6 inhibition may prevent damaged cells from re-entering the cell cycle while repair remains incomplete. Thus, translation of this strategy should consider not only drug selection and CNS exposure, but also treatment timing relative to radiation for treatment during the most susceptible period for the tumor. This distinction is clinically significant because the same schedule that maximizes tumor radiosensitization may also alter normal-tissue toxicity. Experience outside GBM has reported increased toxicity with concurrent CDK4/6 inhibition and RT, emphasizing the need for prospective schedule and safety evaluation rather than assuming that greater antitumor activity will translate directly into a favorable therapeutic index [88].

### 5.2. CDK4/6 Inhibition Combined with Temozolomide

TMZ is an attractive partner because alkylator-induced DNA damage and CDK4/6-mediated cell-cycle control act through complementary mechanisms. Using an intracranial GBM xenograft model, Raub et al. showed that abemaciclib achieved greater brain exposure than palbociclib and, when combined with TMZ, produced additive or greater-than-additive survival benefit compared with either monotherapy. However, these experiments did not establish pharmacologic synergy using formal combination modeling, and the survival benefit should not be interpreted as definitive evidence of synergy. More recently, the brain-penetrant CDK4/6 inhibitor AU3-14 enhanced TMZ antitumor efficacy in orthotopic GBM models and delayed the emergence of TMZ resistance [28]. These data strengthen the rationale for pairing a CNS-optimized CDK4/6iwith standard chemotherapy rather than simply adding a poorly penetrant agent to TMZ. Unlike the RT studies, the optimal sequence of TMZ and CDK4/6 inhibition has not been defined in the literature. This may be biologically important because sustained G1 arrest could either prevent damaged cells from re-entering the cell cycle or, depending on timing and duration, transiently protect a viable drug-tolerant population from DNA-damaging therapy. Thus, schedule dependence is supported most clearly for RT, whereas for TMZ, the available data are insufficient to determine whether concurrent, preceding, or delayed CDK4/6 inhibition is optimal. Future studies should therefore incorporate longitudinal assessment of apoptosis, senescence, and regrowth after treatment withdrawal rather than relying only on short-term proliferation assays.

### 5.3. RTK/PI3K/AKT/mTOR Combination Strategies

Adaptive signaling provides one of the clearest mechanistic rationales for targeted combinations. Palbociclib can produce rebound mTOR activation in GBM and co-treatment with everolimus can block this escape response, disrupt tumor metabolism, increase apoptosis, and significantly prolong survival in orthotopic intracranial models. Everolimus also increased brain concentrations of palbociclib, providing both a biologic and PK basis for the combination [70]. Notably, this interaction was mechanistically linked to reciprocal pathway feedback and increased apoptosis, providing stronger evidence for true synergy than combinations supported only by greater tumor-growth inhibition. Similarly, CDK4/6 inhibition activates c-MET and TrkA/B through NF-kB-dependent induction of HGF, BDNF, and NGF, and combining abemaciclib with the c-MET/Trk inhibitor altiratinib suppressed this adaptive signaling while also having a synergistic effect on apoptosis in GBM models [72].

The PI3K axis has gained additional support from newer patient-derived models. A 2026 study using GBM organoids, primary GBM cells, and xenografts revealed that PI3K induced compensatory CDK4/6 activation, whereas concurrent paxalisib and ribociclib produced synthetic lethality, persistent replication stress, and genomic instability [17]. ERK/MAPK signaling also intersects with this resistance network, where PTEN expression increases palbociclib sensitivity partly through suppression of AKT and ERK signaling [71], and mTOR blockade itself can trigger ERK activation [70]. Thus, direct CDK4/6 plus ERK inhibition is mechanistically attractive, although the GBM-specific preclinical evidence is limited compared to that for mTOR or c-MET/Trk combinations.

These studies also highlight an important distinction from the RT data, which is that most RTK/PI3K/mTOR combinations have been evaluated as concurrent dual blockade rather than through systematic sequence comparisons. Thus, their rationale rests primarily on prevention of adaptive signaling during CDK4/6 inhibition, while the optimal duration of dual treatment and whether sequential therapy can retain efficacy with less toxicity remain largely undefined and should be a matter of future investigations.

### 5.4. Other Rational Combinations

EGFR-directed combinations are increasingly compelling because EGFR alterations frequently coexist with CDKN2A loss or CDK4/6 pathway activation [89,90]. Liu et al. showed that palbociclib plus erlotinib synergistically suppressed GBM proliferation, reduced phosphorylated RB and 4E-BP1, and increased apoptosis in vitro [91]. More recently, temporal kinome profiling of EGFRvIII-driven GBM identified treatment-induced CDK6 upregulation as an adaptive vulnerability and upfront abemaciclib plus neratinib significantly prolonged survival in orthotopic models compared with EGFR inhibition alone [89]. This newer work supports a broader strategy of targeting adaptive kinome rewiring rather than selecting combinations solely from baseline genomic alterations.

Other combinations remain promising but are earlier in development. Dual EZH2 and CDK4/6 inhibition with GSK126 and abemaciclib showed synergy in GBM spheroids, patient-derived organoids, and in ovo models, with mitochondrial dysfunction, replication stress, reduced invasion, and altered stemness programs [92]. Senolytic strategies are also conceptually important because palbociclib can induce a reversible senescence-like quiescence in patient-derived glioma stem cells rather than irreversible senescence [54]. A recent GBM study by Zhang et al. demonstrates that therapy-induced senescent cells can be therapeutically eliminated with senolytic approaches, including clofoctol after TMZ-induced senescence, although direct CDK4/6-senolytic combinations remain to be established [93]. Finally, CDK4/6 inhibition has been shown to influence antitumor immune responses in non-GBM models, which has prompted interest in combining these agents with immunotherapy [94]. Specifically, recent work in glioma provides an additional, although indirect, rationale for this approach. Wang et al. found that USP14 stabilizes PARP1 and, in doing so, suppresses MIC-A/B expression. Moreover, inhibition of USP14 restored MIC-A/B–NKG2D-dependent CD8+ T-cell killing and improved the activity of PD-1 blockade in intracranial glioma models [95]. Khan et al. further highlighted the broader therapeutic relevance of ubiquitin-specific proteases in regulating disease-associated signaling and immune-inflammatory pathways [96]. These findings are intriguing in the context of CDK4/6-directed therapy, but neither study tested CDK4/6 inhibition directly. Accordingly, the USP14–PARP1–MIC-A/B axis should be viewed as a potential mechanistic link to immune-mediated tumor-cell killing rather than as established evidence for a CDK4/6–immunotherapy combination in GBM. Whether CDK4/6 inhibition can modulate this pathway or enhance MIC-A/B-dependent CD8+ T-cell activity will require direct experimental testing. Overall, the most persuasive strategy is not indiscriminate combination therapy, but mechanism-driven pairing of CNS-active CDK4/6 inhibitors with treatments that exploit DNA damage or prevent predictable adaptive escape. These combinations, in addition to some other mechanism-driven combinations, can be found in Table 3.

## 6. Clinical Experience with CDK4/6 Inhibition in Glioblastoma

While clinical experience with CDK 4/6i remains relatively limited with modest results, it has expanded across multiple agents. To date, both approved and investigational CDK4/6i have been evaluated in different phases of clinical trials, with variable efficacy. The following section reviews the clinical experience with individual CDK4/6i. The studies completed to date include monotherapy efficacy trials, presurgical phase 0 PK/PD studies, molecularly selected platform trials, and mechanism-based combination studies, while several abemaciclib studies remain active but no longer actively recruiting. Because these trials differ substantially in disease setting, molecular selection, and primary endpoints, negative or inconclusive results should not be assumed to reflect a single mechanism of failure. Where possible, the discussion below distinguishes inadequate CNS exposure, insufficient target engagement, unfavorable tumor biology, toxicity, and limitations of biomarker selection. Table 4 provides a more detailed comparison of trial status, patient selection, treatment setting, outcome measures, and available results.

### 6.1. Palbociclib

Clinical evaluation of palbociclib provided the first direct test of selective CDK4/6 inhibition in recurrent GBM. In the completed phase II study reported by Taylor et al. (NCT01227434), 22 adults with recurrent RB1-proficient GBM were treated with palbociclib, including a presurgical cohort that permitted paired PK and PD assessment [13]. Unfortunately, the study was terminated early for futility: 95% of evaluable patients progressed within 6 months, median PFS was 5.14 weeks, and median OS was 15.4 weeks. Importantly, several surgical specimens contained drug concentrations considered sufficient for biological activity, yet consistent suppression of RB signaling or proliferation was not demonstrated. These findings argue against inadequate tumor delivery as the sole explanation for failure, but they do not distinguish definitively between incomplete target inhibition, intrinsic tumor resistance, and inadequate biomarker selection. RB positivity alone therefore appears insufficient to identify CDK4/6-dependent GBM [13].

More recently, palbociclib was incorporated into a molecularly matched treatment strategy. In the phase 1/2a N^2^M^2^ umbrella trial, patients with newly diagnosed, MGMT unmethylated GBM were assigned based largely on CDK4/CDK6 amplification or CDKN2A loss and treated concurrently with RT. Palbociclib was administered with RT followed by adjuvant treatment. The palbociclib arm did not meet its prespecified efficacy endpoint, with PFS-6 of 24.4%, median PFS of 4.0 months, and median OS of 12.6 months. Dose escalation also showed that 125 mg with RT produced excessive toxicity, while 100 mg was considered the more tolerable combination dose [109]. Thus, the negative result may reflect several factors, including toxicity-related dosing constraints and the limited predictive value of selecting patients primarily on pathway alterations, rather than a definitive failure of CDK4/6 inhibition itself. Importantly, this study established the feasibility of integrating prospective molecular profiling with biomarker-directed treatment assignment in newly diagnosed GBM patients and echoes the need for more precise determinants of CDK4/6 dependency beyond the conventional pathway alterations.

A similar precision medicine approach is currently being explored in recurrent GBM in the ongoing NCT05432518 pilot study. In this prospective trial, the investigators are molecularly profiling recurrent GBM tissue obtained at resection and then assigning patients to one of several targeted therapies according to the identified tumor alterations. Palbociclib is being administered to patients whose tumor harbors CDK4 or CDK6 amplification, providing an opportunity to test whether more stringent genomic selection can identify a subset of greater susceptibility to CDK4/6 inhibition. This study is still ongoing and efficacy reports are not available at this time [112].

### 6.2. Ribociclib

Ribociclib studies shifted the emphasis from conventional efficacy testing toward prospective demonstration of tumor exposure and target engagement. In a completed phase 0 study of recurrent GBM selected for intact RB together with CDKN2A loss or CDK4/6 amplification (NCT02933736), 12 patients received preoperative ribociclib; six met predefined PK/PD criteria and were entered into an expansion cohort [14]. Tumor exposure and target engagement were confirmed, including RB phosphorylation and proliferation, yet median PFS in the expansion cohort was only 9.7 weeks [14]. A separate phase Ib trial reported tumor penetration in three recurrent GBM patients and similarly confirmed PD activity [58]. The registered protocol also allowed anaplastic glioma, but the published analysis cited here was limited to recurrent GBM. Collectively, these studies provide relatively strong evidence that inadequate CNS penetration alone does not explain the limited activity of ribociclib. Rather, they suggest that effective CDK4/6 target engagement may be insufficient in tumors with parallel or adaptive survival pathways. However, because these were small phase 0/PK-PD studies and were not powered primarily for clinical efficacy, the relative contributions of tumor biology and biomarker selection cannot be resolved definitively. The observation of compensatory PI3K/mTOR activation following ribociclib treatment subsequently motivated evaluation of combined CDK4/6 and mTOR inhibition. In the completed phase 0/1 trial NCT03834740, 24 patients with recurrent high-grade glioma harboring CDKN2A/B deletion or CDK4/6 amplification, additional PI3K/mTOR pathway alterations, and intact Rb, received preoperative ribociclib in combination with everolimus. While ribociclib achieved pharmacologically relevant concentrations intracerebrally, including non-enhancing regions of the tumor, unbound everolimus concentrations remained below the limit of detection in the tumor tissue. Even though ribociclib exposure reduced MIB1/Ki-67 labeling, suggesting cell-cycle inhibition, the poor intratumoral exposure of everolimus limited the ability of this regimen to adequately test dual inhibition of mTOR and CDK 4/6 [57]. Thus, this study did not provide a definitive clinical test of the biological combination because adequate dual-pathway exposure was not achieved. The inconclusive efficacy of the regimen is more appropriately attributed to inadequate exposure of the combination partner than to failure of the underlying mTOR-resistance hypothesis.

### 6.3. Abemaciclib

Abemaciclib has undergone the broadest GBM-specific clinical development. Early first-in-human experience reported by Patnaik et al. included a GBM cohort and provided the initial clinical rationale for dedicated studies [59]. In the multicenter phase 0/II recurrent GBM trial NCT02981940, patients were selected for enriched CDKN2A/B loss or CDK4/6 amplification with intact RB. The trial remains active but is no longer recruiting, with study completion currently expected in 2027. Interim results from the nonsurgical cohort showed limited single-agent activity, with a 6-month PFS rate of 9.37%, median PFS of 55 days, and median OS of 384 days. As per the latest reports from September 2025, 45 patients had been enrolled in this study and 42 had been treated [66]. Because the study has demonstrated that abemaciclib can reach GBM tissue, the modest efficacy is unlikely to be explained solely by failure of CNS delivery. However, the available data cannot distinguish conclusively between incomplete pathway dependence, adaptive resistance, and insufficient molecular selection based on canonical RB-pathway alterations.

A different signal emerged in newly diagnosed GBM through the randomized adaptive INSIGhT platform (NCT02977780). Among 237 treated patients with newly diagnosed MGMT-unmethylated GBM, 73 were in the abemaciclib arm. Abemaciclib was associated with longer PFS relative to the shared control arm (HR 0.72; one-sided *p* = 0.046), but no significant OS benefit was observed [60]. The abemaciclib arm is now closed to accrual, although the broader INSIGhT platform remains active. The contrast between recurrent-disease monotherapy and the PFS signal in newly diagnosed GBM suggests that disease setting, prior treatment pressure, and combination with frontline therapy may influence the clinical value of CDK4/6 inhibition. However, because the studies differed in design, population, and treatment context, these data cannot establish that treatment history itself accounts for the difference in outcome.

Clinical development for abemaciclib has increasingly moved toward mechanism-based combinations and biomarker-driven designs. The completed phase 0/2 trial NCT04391595 evaluated abemaciclib plus the ERK1/2 inhibitor LY3214996 in recurrent GBM selected for intact RB, elevated pERK, and CDKN2A/B deletion or CDK4/6 amplification. Forty-two patients were treated in the phase 0 component and a 2024 report showed pharmacologically relevant abemaciclib exposure in non-enhancing tumor in 80% of patients but adequate LY3214996 exposure in only 30%. Only 22.5% met the dual-drug PK trigger, and the reported phase II median PFS and OS were 2.4 and 7.2 months, respectively [87]. Unlike several earlier trials, this study provides direct evidence that inadequate exposure and target modulation of the combination partner drug contributed to the limited efficacy. Even here, however, the trial cannot establish that inadequate ERK inhibition was the only mechanism of treatment failure.

Other GBM-specific efforts have been more exploratory. For instance, a planned phase II study of abemaciclib plus pembrolizumab in recurrent GBM (NCT04118036) was withdrawn before enrollment and provides no efficacy, toxicity, or mechanistic information [98]. In a separate pilot study, the authors evaluated abemaciclib plus bevacizumab in recurrent GBM selected for CDKN2A/B loss or CDK 4/6 gain/amplification (NCT04074785). Among the nine treated patients, median PFS was 14 weeks and median OS was 20 weeks. While the combination treatment was found to be feasible, the preliminary analysis did not demonstrate a clear advantage in survival outcomes over the historical experience with using bevacizumab alone [110]. NCT03220646, a phase II basket study of abemaciclib in recurrent primary brain tumors that includes a molecularly defined GBM cohort, remains active but is no longer recruiting. GBM-specific efficacy results remain insufficiently reported to draw firm conclusions [108].

## 7. Strategies for Biomarker and Ideal Patient Selection

The central translational challenge for CDK4/6 inhibition in GBM is no longer establishing the frequency of pathway alteration, but identifying the tumors in which CDK4/6 signaling represents a true therapeutic dependency. The TCGA established that disruption of the RB-cell-cycle axis is among the most common genomic events in GBM, yet clinical experience till date has shown that pathway alteration alone is a poor surrogate for therapeutic addiction [10]. This is precisely the setting in which a predictive biomarker becomes important. Unlike a prognostic marker, which is associated with outcome independent of a specific therapy, a predictive biomarker is intended to identify patients more likely to benefit from a particular treatment [113,114]. In oncology, biomarkers can guide best treatment selection, but their clinical value depends on meaningful treatment response rather than simply addressing the underlying biology or aggressiveness of the disease [115]. This is a prevalent challenge in GBM because marked inter- and intratumoral molecular heterogeneity can complicate biomarker validation and contribute to failure of single-target therapeutic strategies as seen historically [116].

Broadly speaking, a clinically meaningful biomarker should have a clear biological rationale, a reproducible and analytically reliable assay, clinical validity in the target population, and evidence of clinical utility such that use of the biomarker can meaningfully improve treatment selection or drug development decisions [113,114,115,117]. For targeted agents, this also means that the biomarker should reflect the mechanism of drug sensitivity or resistance closely enough to distinguish true pathway dependence from the mere presence of a pathway alteration from genomic studies [117,118]. Thus, a reliable biomarker strategy should include identifying tumors that satisfy three criteria: an intact downstream target required for response, evidence of functional dependence on CDK4/6 signaling, and absence of dominant compensatory bypass pathways that can sustain E2F activity despite CDK4/6 blockade. Because CDK4/6i sensitivity and resistance can be shaped by multiple nodes within and outside the canonical RB pathway, a composite biomarker is more biologically plausible than reliance on any single alteration [118].

RB1 status should therefore be considered a necessary starting point for patient selection rather than a sufficient biomarker on its own. Additional molecular features may help identify tumors that are more likely to remain dependent on CDK4/6 signaling, although the strength of evidence varies considerably. CDKN2A/B loss and CDK4/6 amplification have already been used as eligibility or enrichment criteria in clinical studies, but neither has been prospectively validated as a stand-alone predictor of clinical benefit [13,14]. Cyclin D expression, CDK6 activity, proliferation indices such as Ki-67, and E2F-associated transcriptional signatures may provide additional information about pathway activity, but these remain largely exploratory [11,24]. By contrast, treatment PD changes, including suppression of phospho-RB and reduction in Ki-67 after short-term drug exposure, may provide a more direct measure of whether the pathway has been effectively engaged in a given tumor [14]. Future clinical trials may be better served by a composite approach that incorporates RB competence, evidence of pathway activation, assessment of major bypass mechanisms, and confirmation of target engagement rather than relying on a single genomic alteration. Finally, in GBM, the additional problem of molecular heterogeneity further supports a multidimensional approach rather than selection based on one genomic feature alone [116].

RB1 integrity is the most defensible first selection criterion. RB-deficient GBM models are intrinsically resistant to palbociclib, whereas RB-proficient models undergo G1 arrest [12]. However, RB positivity is necessary rather than sufficient. For instance, the phase II palbociclib study restricted enrollment to RB1-proficient recurrent GBM but was terminated for lack of efficacy [13]. Similarly, phase 0 ribociclib studies selected tumors with an intact RB pathway and demonstrated tumor exposure and PD activity without durable clinical benefit [24,58]. Thus, RB1 should function as an exclusionary gate, with loss, mutation, or functional absence precluding CDK4/6 monotherapy. However, RB positivity alone should not define eligibility.

Upstream activation markers may identify tumors more dependent on the pathway, but they require contextual interpretation. CDKN2A/B loss, p16 loss, CDK4 amplification, CDK6 upregulation, and CCND alterations all provide a mechanistic rationale for CDK4/6 blockade [10]. In the 2026 study by Bueno et al., CDKN2A/B loss was substantially more frequent in short-term than long-term GBM survivors after aggressive resection (83.3% vs. 43.8%), and EGFR amplification was also enriched in short-term survivors, suggesting that these alterations can mark biologically aggressive disease rather than drug sensitivity [90]. A 2026 TCGA-informed analysis likewise demonstrated the heterogeneous distribution of CDKN2A/B, CDK4, CDK6, and RB1 alterations across GBM [119]. These findings reinforce the distinction between a prognostic marker and a predictive biomarker.

Population-level genomic structure should also be treated as a trial-design variable rather than a background characteristic. Cimino et al. compared copy-number subtype distributions across TCGA, the German Glioma Network, the ARTE trial in elderly patients, and a paired initial/recurrent GBM cohort and found that clinically selected cohorts were enriched for copy-number profiles associated with longer survival. This demonstrates that the molecular composition of trial populations can differ systematically from unselected GBM populations, creating an important source of confounding when evaluating biomarker-enriched targeted therapies [120]. For CDK4/6 studies, balancing or stratifying patients by copy-number architecture may be particularly relevant because CDK4 amplification, CDKN2A/B loss, MDM2 co-amplification, and related cell-cycle alterations can occur within broader genomic subtypes and may influence prognosis independently of treatment response.

Related work using multidimensional scaling of diffuse gliomas showed that integrated DNA-level molecular signatures identify prognostically distinct subgroups beyond conventional histology, supporting multidimensional rather than single-marker stratification [121]. Ene et al. highlighted patient-specific genomic features, including CDK4/MDM2 co-amplification, and argued that genomic signatures could inform clinical trial design and treatment stratification [122]. Together, these studies suggest that future CDK4/6 trials should distinguish predictive biomarkers of drug sensitivity from molecular features that primarily define prognosis or trial-enrollment bias. A practical design would therefore stratify by age and global copy-number or molecular subtype while separately testing functional biomarkers of CDK4/6 dependence.

Additional functional markers may be more informative. Proneural glioma stem-cell models with intact RB1, higher CDK6 expression, and an E2F-associated transcriptional program were preferentially sensitive to palbociclib, whereas RB-null/CDK4-overexpressing models were resistant [24]. Moreover, PTEN may further refine selection based on the findings from the study by Liu et al. that showed that restoration of PTEN increased palbociclib sensitivity through suppression of AKT and ERK signaling [71]. Conversely, more recently, Zhang et al. demonstrated that PI3K and CDK4/6 co-activation can sustain tumor growth and that concurrent inhibition is synthetically lethal, suggesting that PI3K-active tumors may be better candidates for combination therapy than CDK4/6i monotherapy [17]. Based on Lin et al.’s work, EGFR may similarly guide combinations. Adaptive kinome profiling of EGFRvIII-driven GBM showed treatment-induced CDK6 accumulation and improved survival when EGFR and CDK4/6 were inhibited together [89].

Recent functional genomics adds potentially stronger negative and combination biomarkers. Genome-wide CRISPR screening in glioma identified CCNE1 gain and AMBRA1 loss as mediators of reduced CDK4/6 inhibitor sensitivity, while CHEK1 or FAM122A disruption created synthetic-lethal interactions with CDK4/6 inhibition [99]. These findings are consistent with broader dependency analyses in which high RB1/CCND1 and low CCNE1 expression characterized CDK4/6-sensitive states, whereas cyclin E-CDK2 dependence characterized resistance [123]. An additional emerging marker is the stem-like/mesenchymal transcription factor tGLI1. Recent work from our lab has associated high tGLI1 with CDK4/6 inhibitor resistance and showed sensitization after tGLI1 inhibition [75].

Biomarker development in GBM may also need to explore beyond conventional cell-cycle markers. Zhu et al. identified ADGRG6 as a prognostic and immune-associated marker across cancers, with glioma data linking higher expression to adverse outcomes and NF-κB-related tumor biology [124]. Although this was not a study of CDK4/6 inhibition, it illustrates how tumor-intrinsic molecular programs may also reflect the immune context of the tumor. Ramalingam et al. similarly used transcriptomic and network-based analyses to identify drug-resistance biomarkers in another tumor model, supporting the broader value of pathway-level signatures rather than reliance on single genes alone [125]. These types of exploratory molecular and immune-contexture features could eventually complement RB status and CDK4/6 pathway alterations in future GBM biomarker studies.

**Figure 6:**
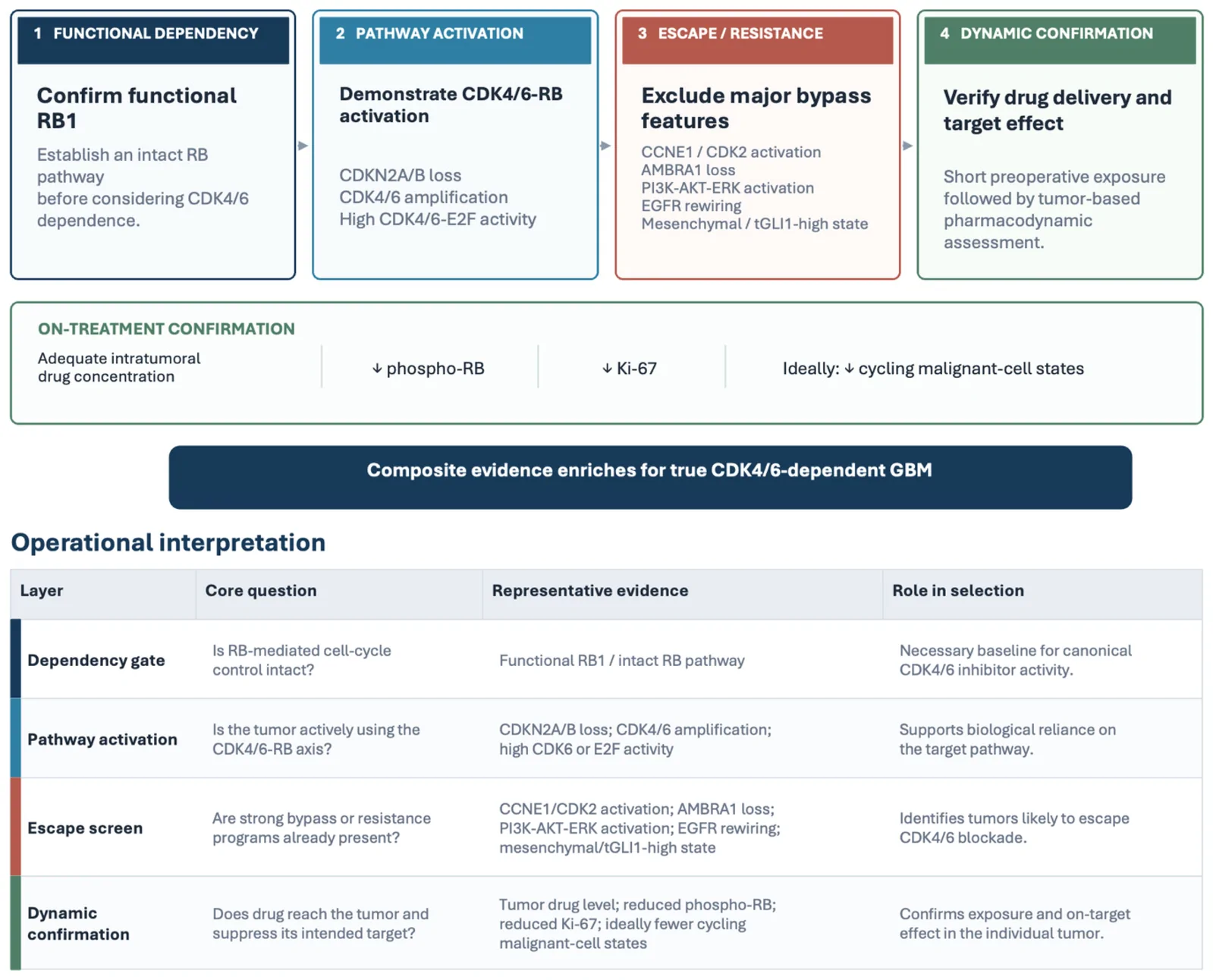
Proposed biomarker framework for identifying CDK4/6-dependent glioblastoma. A stepwise selection strategy is proposed to select for glioblastoma patients most likely to depend on the CDK4/6-RB axis. First, functional RB1 integrity should be established as a prerequisite for canonical CDK4/6 dependence. Second, evidence of pathway activation, including CDKN2A/B loss, CDK4/6 amplification, or elevated CDK4/6-E2F activity, can support biologic reliance on the pathway. Third, major escape and resistance mechanisms including CCNE1/CDK2 activation, AMBRA1 loss, PI3K-AKT-ERK signaling, EGFR rewiring, and mesenchymal or tGLI1-high cell states, should be assessed to identify tumors with a high likelihood of bypassing CDK4/6 blockade. Finally, confirmation of drug delivery and target engagement through short preoperative exposure and tumor-based pharmacodynamic assessment can verify adequate intratumoral drug concentration, suppression of phospho-RB and Ki-67, and, ideally, reduction in cycling malignant-cell states. Integration of these complementary layers provides composite evidence for true CDK4/6 dependence and may support more rational patient selection for CDK4/6-directed therapy in GBM.

Thus, taking this all together, a practical future strategy should use layered selection rather than a single mutation. First, confirm functional RB1. Second, document pathway activation (for example, CDKN2A/B loss, CDK4/6 amplification or high CDK6/E2F activity). Third, screen for escape features such as CCNE1/CDK2 activation, AMBRA1 loss, PI3K/AKT/ERK activation, EGFR-driven rewiring, or mesenchymal/tGLI1-high states. Finally, incorporate a dynamic biomarker: a short preoperative exposure followed by demonstration of adequate intratumoral drug concentration, decreased phospho-RB, reduced Ki-67, and ideally a reduction in cycling malignant-cell states [14,24,53,57,58]. Such a composite genomic-transcriptomic-PD approach is more likely to identify true CDK4/6-dependent GBM than conventional eligibility based on RB1 or CDKN2A status alone (Figure 6). Table 5 summarizes the candidate biomarkers and patient-selection strategy for CDK4/6 inhibition in GBM.

## 8. Discussion: Why Has Translation Failed and How Do We Move Forward?

The clinical experience with CDK4/6 inhibition in GBM has exposed a central paradox: while the cyclin D-CDK4/6-RB axis is frequently dysregulated, pharmacologically targetable, and clearly important to tumor biology, CDK4/6is have produced little durable clinical benefit in GBM. The explanation is not a single failed drug or target, but a convergence of pharmacologic, biologic, and trial-design limitations.

Inadequate drug delivery remains the first barrier. As discussed above, palbociclib is strongly restricted by ABCB1/P-glycoprotein and ABCG2/BCRP, while ribociclib and abemaciclib achieve greater but still heterogeneous CNS exposure [45,46,47]. Phase 0 studies have shown that drug concentrations can differ between enhancing and non-enhancing tumors and that measurable tissue exposure does not necessarily translate into durable benefit [14,52,53]. This argues for prioritizing unbound intratumoral concentration and PD target suppression rather than plasma or CSF exposure alone. CNS-optimized agents such as AU3-14 represent an important next step, but improved penetration must ultimately be demonstrated in infiltrative tumors before efficacy can be reliably assumed [28].

GBM heterogeneity and incomplete pathway dependence provide a second explanation. RB1 integrity is necessary for canonical response, but clinical trials demonstrate that RB proficiency alone is insufficient [13]. CDKN2A/B loss, CDK4 amplification, CDK6 expression, EGFR signaling, PI3K activation, and E2F output occur in different combinations and cellular states. This produces variable degrees of CDK4/6 dependence. The N^2^M^2^ umbrella trial further illustrates the broader challenge of translating molecular matching into efficacy in GBM, even when targeted agents are assigned according to tumor alterations [109]. Future selection should therefore move from single-gene eligibility toward composite genomic, transcriptomic, and functional biomarkers. The translational confidence of individual strategies should be interpreted in the context of the models used, particularly because many preclinical studies do not reproduce the treatment history, infiltrative growth, and evolving cellular states of recurrent GBM.

A third limitation is the predominantly cytostatic nature of CDK4/6 inhibition rather than being cytotoxic. Reversible drug-tolerant persistence has been demonstrated in patient-derived glioma stem cells, which can resume proliferation after palbociclib withdrawal [54]. Strategies that convert arrest into apoptosis or selectively eliminate persister populations, including rational DNA-damage combinations and emerging senolytic approaches, deserve further evaluation [31,89].

Fourth, GBM rapidly activates compensatory signaling. Rebound PI3K/AKT/mTOR activity, c-MET/Trk signaling, EGFR-associated kinome rewiring, and transcriptional plasticity can restore survival or proliferation despite CDK4/6 blockade [17,64,66,72]. The implication is that combinations should be selected from mechanistic evidence of adaptive escape rather than added empirically. Importantly, every component of a combination must independently achieve adequate concentrations within the tumor, as illustrated by phase 0 studies in which successful CDK4/6 target engagement was undermined by insufficient delivery of the agent [80].

Finally, early studies have generally used biomarkers that identify pathway alteration more reliably than pathway addiction. Contemporary analyses show that molecular selection remains inconsistently incorporated into recurrent GBM trials, even though CDKN2A/B, CDK4/6, EGFR, and RB are among the most frequently investigated candidate markers [126]. More recent precision-medicine studies demonstrate that recurrent-tumor profiling and multiagent regimens can be operationalized, including approaches in which abemaciclib forms part of a genomically tailored combination [127]. These strategies are not yet evidence that precision CDK4/6 therapy improves survival, but they support a more dynamic model of patient selection than reliance on archival tissue or a single genomic lesion.

Collectively, future success is unlikely to come from indiscriminate monotherapy with a first-generation CDK4/6i. A more rational development pathway would integrate a CNS-optimized compound, confirmation of adequate intratumoral concentrations, demonstration of RB-pathway suppression, composite biomarker selection, and co-targeting of the dominant adaptive escape mechanism. Future trials should also incorporate longitudinal tissue, imaging, and circulating biomarkers whenever feasible, because treatment itself may alter cellular state and pathway dependence and make treatment a more dynamic than static process. CDK4/6 remains a compelling therapeutic node in GBM, but its value will depend on treating it as one component of a dynamic, heterogeneous signaling network rather than as an isolated cell-cycle target.

## 9. Conclusions

The central question addressed in our review is why a pathway so frequently dysregulated and biologically important in GBM has produced only modest clinical benefit when therapeutically inhibited. The evidence to date supports a strong biological rationale for targeting CDK4/6 in GBM, with substantial activity in preclinical models and early clinical studies showing that selected agents can reach tumor tissue and inhibit the intended pathway. What has not yet been shown is a clear survival benefit for patients. Heterogeneous CNS drug exposure, variable dependence on the cyclin D-CDK4/6-RB axis, adaptive signaling, intratumoral heterogeneity, cell-state plasticity, and reversible cytostasis have all emerged as major determinants of response.

The remaining challenge is therefore not simply whether CDK4/6 can be inhibited, but whether a given tumor is truly dependent on the pathway, whether functional RB is retained, whether adaptive resistance can be controlled, and whether adequate drug exposure can be achieved across the heterogeneous and infiltrative tumor compartment. These barriers are substantial, but they are now better defined than they were when CDK4/6 inhibitors first entered GBM studies. With more selective patient enrollment, CNS-optimized compounds, confirmation of intratumoral target engagement, and combinations directed at specific resistance mechanisms, there remains a realistic opportunity to translate the strong biological rationale for CDK4/6 inhibition into meaningful clinical benefit for a subset of patients with GBM.

## Figures and Tables

**Table 1:** Comparative Implications of CDK4/6-RB Pathway Alterations for CDK4/6 Inhibitor Response in Glioblastoma.

Molecular Alteration	Functional Consequence	Predicted Response to Selective CDK4/6 Inhibition	GBM-Specific Evidence/Interpretation	Reference
RB1 loss, mutation, or functional inactivation	Eliminates the downstream RB checkpoint required for CDK4/6 blockade to restrain E2F-mediated G1/S progression.	Intrinsic resistance	RB-deficient GBM cell lines were resistant to palbociclib, whereas RB-proficient models underwent G1 arrest. Experimental RB depletion conferred resistance, supporting functional RB as a prerequisite for canonical CDK4/6 inhibitor activity.	[12]
CDKN2A/B homozygous deletion/p16 loss with intact RB1	Removes endogenous p16-mediated inhibition of CDK4/6 while preserving the downstream RB target, potentially increasing reliance on CDK4/6 signaling.	Potential sensitivity/enrichment phenotype; not sufficient alone	In patient-derived GBM xenografts, CDKN2A/B-deleted tumors with wild-type RB and nonamplified CDK4 were among the most sensitive to palbociclib. Thus, p16 loss may indicate greater CDK4/6 dependence when RB remains functional.	[11]
CDK4 amplification with intact RB1	Increases CDK4 dosage and pathway activity; may occur within broader oncogenic amplicons and does not necessarily establish CDK4/6 addiction.	Context-dependent; not a reliable sensitivity marker and may associate with resistance	Cen et al. identified CDK4-amplified, p16-retaining GBM xenografts that were resistant to palbociclib. CDK4 amplification therefore should not be equated with CDK4/6 inhibitor sensitivity.	[11,18]
CDK6 amplification/high CDK6 expression with intact RB1	Enhances CDK6-mediated RB phosphorylation and may increase pathway dependence in selected transcriptional or cellular states.	Potential sensitivity, but strongly context-dependent	A CDKN2A/B-deleted GBM xenograft with high-level CDK6 amplification was sensitive to palbociclib. Proneural glioma stem-like models with intact RB and higher CDK6 expression also showed greater sensitivity to CDK4/6 inhibition.	[11,24]
Intact RB1 without a clear upstream activating alteration	Preserves the molecular machinery required for drug-induced RB hypophosphorylation but does not demonstrate pathway addiction.	Necessary but insufficient biomarker	A phase II palbociclib study restricted enrollment to RB1-positive recurrent GBM yet demonstrated limited clinical activity, showing that RB positivity alone is insufficient for patient selection.	[13]
Intact RB1 with CDKN2A loss and/or CDK4/6 amplification	Combines preservation of the downstream RB effector with evidence of upstream pathway activation.	Biologically enriched population, but clinical sensitivity remains uncertain	In a phase 0 ribociclib trial, selection for intact RB plus CDKN2A deletion and/or CDK4/6 amplification produced measurable intratumoral drug exposure and suppression of RB phosphorylation, but median PFS remained short. Genomic enrichment and target engagement therefore did not ensure durable response.	[14]
CDKN2A loss plus RB1 loss/dysfunction	Removes both the upstream p16 brake and the downstream RB effector.	Predicted resistance despite CDKN2A loss	The potentially sensitizing implication of CDKN2A loss depends on retention of functional RB. Loss of RB removes the principal downstream mediator of CDK4/6 inhibitor-induced cell-cycle arrest.	[12,22]
CDK4 amplification plus p16 retention	Represents strong CDK4 pathway activation despite persistence of the endogenous p16 brake and may reflect a pathway configuration that is not fully suppressible by standard CDK4/6 blockade.	Possible resistance phenotype	In the Cen et al. panel, CDK4-amplified, p16-positive GBM xenograft models were resistant to palbociclib, providing direct evidence that CDK4 amplification alone is not a positive predictive biomarker.	[11]

**Table 3:** Combination Drug Strategies Based on Preclinical evidence.

Combination	Scientific Rationale	Treatment Agent(s)	Model/Setting	Principal Finding	Resistance Mechanism	Translational Maturity	Treatment Schedule/Sequence	Evidence for Interaction	References
CDK4/6i + RT	Impair post-RT repair/regrowth	Palbociclib	RB-intact intracranial xenografts	Delayed DNA repair and prolonged regrowth/survival	DNA damage recovery	Strong GBM preclinical rationale; clinical optimization needed	Concurrent or post-RT superior to pre-RT	Strong schedule-dependent radiosensitization	[12,31]
CDK4/6i + TMZ	Alkylation cell-cycle arrest	Abemaciclib; AU3-14	Orthotopic GBM models	Improved survival; delayed TMZ resistance	TMZ tolerance	Promising preclinical; uncertain clinical exposure	Mostly concurrent; optimal sequence unknown	Improved efficacy; formal synergy not consistently established	[15,28]
CDK4/6i + mTORi	Block rebound mTOR	Palbociclib + everolimus	Cell lines/orthotopic models	More apoptosis and improved survival	mTOR feedback	Preclinical support; everolimus tumor delivery limits translation	Concurrent dual blockade	Strong mechanistic synergy	[70]
CDK4/6i + PI3Ki	Target PI3K/CDK4/6 co-dependence	Ribociclib + paxalisib	Patient-derived GBM cells, organoids, and xenografts	Synthetic lethality; replication stress	PI3K-driven bypass	Preclinical; dual CNS exposure unproven	Concurrent	Synthetic lethality in preclinical models	[17]
CDK4/6i + c-MET/Trki	Block treatment induced RTK activation	Abemaciclib + altiratinib	Glioma-initiating cell, patient-derived cell models and xenografts	Restored sensitivity; increased apoptosis	MET/Trk escape	Preclinical, no efficacy trial	Concurrent	Strong mechanistic synergy	[72]
CDK4/6i + EGFRi	Block EGFR-induced CDK6 rewiring	Palbociclib + erlotinib; abemaciclib + neratinib	GBM cell lines; EGFRvIII orthotopic models	Synergy or improved survival	EGFR/CDK6 adaptive rewiring	Biomarker-specific; preclinical	Concurrent/upfront co-targeting	Preclinical synergy; blocks adaptive CDK6 escape	[89]
CDK4/6i + ERK/MAPKi	Suppress MAPK escape	Abemaciclib + LY3214996	Recurrent GBM phase 0/2	Dual-drug PK/PD often inadequate	ERK bypass	Clinical testing; partner-drug exposure limits inference	Concurrent	Mechanistically plausible; limited GBM-specific evidence	[87,97]
CDK4/6i + EZH2i	Target epigenetic plasticity	Abemaciclib + GSK126; palbociclib + AC1Q3QWB	GBM spheroids, patient-derived organoids, and in vivo models;	Increased antitumor activity,	Transcriptional plasticity; stemness	Preclinical	Concurrent	Preclinical synergy	[92]
CDK4/6i + senolytic strategy	Eliminate arrested persisters	Palbociclib + proposed senolytic agents	Patient-derived GSCs; GBM senescence models and studies	Reversible arrest documented; combination untested	Drug-tolerant persistence	Hypothesis-generating only	Likely sequential	Hypothesis-generating; direct synergy not established	[54]
CDK4/6i + immunotherapy	Potential immune modulation	Abemaciclib + PD-(L)1 blockade	Predominantly non-GBM immune-competent models;	No interpretable GBM efficacy	Immune-suppression	Speculative in GBM	Optimal schedule unknown	Speculative in GBM	[94,98]
CNS-active CDK4/6i + CHK1i	Exploit checkpoint dependency	CDK4/6 inhibitor + CNS-penetrant CHK1 inhibitor	Glioma CRISPR and drug models	Synthetic lethal vulnerability	Checkpoint-mediated persistence	Early preclinical; CNS exposure/toxicity unknown	Concurrent	Synthetic-lethal rationale; preclinical	[99]
CDK4/6i + CDK2/cyclin Ei	Prevent cyclin E-CDK2 bypass	CDK4/6i + selective CDK2/cyclin Ei	Glioma resistance models and other cancers	CCNE1 modifies sensitivity; dual regimen not validated	CDK2/E2F escape	Proposed; no direct GBM regimen evidence	Concurrent/proposed	Strong rationale; limited direct GBM combination data	[99,100,101]
CDK4/6i + MDM2i	Restore p53-mediated death	CDK4/6i + MDM2i	CDK4/MDM2-amplified GBM concept	Proposed arrest-to-apoptosis conversion	Cytostatic persistence	Novel but unvalidated combination	Concurrent/proposed	Rational but not validated	[102,103,104]
CDK4/6i + FAKi	Co-target invasive signaling	CDK4/6i + FAK/paxillin-pathway inhibitor	GBM CDK4/FAKi invasion studies	Invasion mechanism established; combo untested	RB-independent invasion	Novel but not validated	Concurrent/proposed	Mechanistically rational; preclinical evidence remains limited	[105,106]
CDK4/6i i + ALDH1A3-directed therapy	Counter mesenchymal transition	CDK4/6i + ALDH1A3-selective therapy	Patient-derived proneural GSC	Greater growth suppression	Therapy-induced proneural-to-mesenchymal plasticity	Early preclinical; agent availability limits translation	Concurrent/adaptive-state directed	Early preclinical evidence	[24]
CDK4/6 inhibitor + tGLI1-directed therapy	Overcome stemness-linked resistance	CDK4/6i + tGLI1inhibitor	Patient-derived GBM/GSC models and orthotopic studies	Sensitization; more apoptosis	tGLI1-associated resistance	Preclinical; selectivity/interaction concerns with ketoconazole	Concurrent	Early preclinical evidence	[75,107]

**Table 4:** Clinical trials using CDK4/6 inhibitors in glioblastoma patients and their features including outcomes.

Trial Initiation	Agent	Trial/Phase	Trial Status	Population/Molecular Selection	Treatment Strategy	Design/N	Key Outcomes	Key Limitations/Toxicity	References
2010	Palbociclib	NCT01227434; Phase II	Terminated	Recurrent GBM; RB1-positive by IHC; up to 3 prior relapses	Palbociclib 125 mg once daily, days 1–21 of each 28-day cycle; surgical cohort received preoperative drug for PK/PD analysis	n = 22 (6 surgical, 16 nonsurgical)	PFS 5.14 wk; OS 15.4 wk; stopped for futility.	RB1 alone inadequate; drug exposure did not ensure efficacy.	[13]
2015	Ribociclib	NCT02345824; Phase I/Ib surgical PK/PD study	Completed	Recurrent GBM/anaplastic glioma patients undergoing resection; RB-pathway assessment	Preoperative ribociclib followed by postoperative therapy in eligible patients; postoperative dosing generally 600 mg daily, 21 days on/7 days off	Small surgical PK/PD cohort	Ribociclib penetrated recurrent glioma tissue at pharmacologically relevant concentrations and demonstrated evidence of CDK4/6 target engagement. Durable clinical activity was limited.	Small cohort; study designed primarily for PK/PD rather than efficacy.	[58]
2016	Ribociclib	NCT02933736; Phase 0 with expansion	Active, not recruiting	Recurrent GBM with intact RB and CDKN2A deletion and/or CDK4/CDK6 amplification	900 mg daily for 5 days before resection; patients meeting prespecified intratumoral PK/PD criteria could continue postoperative therapy	n = 12 with PK/PD-guided expansion	Good CNS/tumor penetration; Reduced pRB/Ki-67; expansion PFS 9.7 wk; Post-treatment tumors showed compensatory PI3K/mTOR activation.	Four phase-0 patients were ultimately considered to have pseudoprogression; adaptive signaling likely contributed to resistance.	[14]
2016–2017	Abemaciclib	INSIGhT, NCT02977780; randomized Phase II adaptive platform	Recruiting but not abemaciclib arm	Newly diagnosed MGMT-unmethylated GBM; adaptive molecular assignment	Abemaciclib experimental arm after standard RT versus contemporaneous control within the INSIGhT platform	Bayesian adaptive randomized platform	Abemaciclib improved PFS but not OS. Reported median PFS approximately 6.3 vs. 4.6 months (HR ~0.72); no convincing OS benefit.	PFS signal did not translate into improved OS.	[60]
2017–2020	Abemaciclib	NCT02981940; Phase 0/II	Active, not recruiting	Recurrent GBM; CDKN2A/B loss with intact RB	Surgical PK cohort plus continuous oral abemaciclib in nonsurgical efficacy cohort	Reported nonsurgical Phase II cohort n = 32	PFS-6 9.4%; median PFS 55 days; median OS 384 days.	Limited efficacy despite CNS penetration; hematologic toxicity.	[66]
2017	Abemaciclib	NCT03220646; Phase II basket trial	Active, not recruiting	Recurrent primary CNS tumors, including GBM and other histologies	Abemaciclib monotherapy in genomically defined CNS tumor cohorts	Basket study	GBM-specific outcomes not established.	Mixed diagnoses; no definitive GBM efficacy.	[108]
2018	Palbociclib	N^2^M^2^/NOA-20, NCT03158389; Phase I/IIa umbrella trial	Completed	Newly diagnosed MGMT-unmethylated GBM; palbociclib assignment based on CDK4/CDK6 amplification or CDKN2A loss	Palbociclib with RT, then adjuvant palbociclib; 75, 100 and 125 mg dose levels evaluated	Palbociclib efficacy cohort n = 41	Failed prespecified efficacy endpoint: PFS-6 24.4% (10/41), median PFS 4.0 months, median OS 12.6 months.	Endpoint unmet; 125 mg with RT too toxic. Hematologic toxicity predominated.	[109]
2019	Ribociclib + everolimus	NCT03834740; Phase 0/I	Completed	Recurrent high-grade glioma undergoing resection, including recurrent GBM	Preoperative dual blockade	PK/PD study	Ribociclib adequate tumor exposure; everolimus poorly detected.	Primarily a PK/PD study; not powered to establish clinical efficacy.	[57]
2019	Abemaciclib + bevacizumab	NCT04074785; pilot/early Phase I	Terminated	Recurrent GBM with CDKN2A/B loss or CDK4/6 gain/amplification; RB1 intact	Abemaciclib 150 mg BID plus bevacizumab 10 mg/kg every 2 weeks	10 enrolled; 9 received ≥1 cycle	Median PFS 14 weeks and median OS 20 weeks; no clear evidence of synergistic benefit.	Small uncontrolled trial; no clear benefit over historical expectations.	[110]
2019	Abemaciclib + pembrolizumab	NCT04118036; Phase II	Withdrawn	Recurrent GBM at first recurrence	Abemaciclib plus pembrolizumab	Withdrawn	Study was withdrawn; no interpretable GBM efficacy data were generated.	Withdrawn before enrollment. No efficacy conclusions possible.	[98]
2020	Abemaciclib + LY3214996 (temuterkib)	NCT04391595; Phase 0/II	Completed	Recurrent GBM; intact RB, pERK enrichment, and CDKN2A/B deletion or CDK4/6 amplification	Six days of preoperative dual blockade; continuation if prespecified tumor PK criteria met	n = 42; 40 evaluable for PK	Dual PK target met by 22.5%; PFS ~2.4 mo; OS ~7.2 mo.	ERK partner exposure/PD inadequate.	[87]
2021	Auceliciclib (AU3-14) + TMZ	ATTACK-1; ACTRN12621000479808; Phase I/IIa	Completed	Advanced solid tumors followed by recurrent/relapsed; high grade glioma cohort	Auceliciclib monotherapy dose escalation followed by auceliciclib plus temozolomide; recommended combination reported as 300 mg BID + TMZ 100 mg daily	HGG cohort n = 17	Preliminary disease control in 3 HGG patients; Preliminary activity only.	Small heterogeneous cohort; clinical benefit unproven.	[111]
2022	Palbociclib as matched therapy	NCT05432518; early precision-medicine trial	Recruiting	Recurrent GBM undergoing surgery; treatment selected from ex vivo/molecular profiling; palbociclib used for appropriate CDK4/6-pathway alterations	palbociclib generally 125 mg daily when molecularly indicated	Pilot	No mature drug-specific efficacy results available as of August 2026.	Small multiagent precision-design study; isolating palbociclib-specific efficacy will be difficult.	[112]

**Table 5:** Candidate Biomarkers and Patient-Selection Strategy for CDK4/6 Inhibition in GBM.

Biomarker	Biological Implication	Evidence	Clinical Interpretation	Assay Strategy	Role	References
Functional RB1/pRB axis	Required downstream target for CDK4/6 blockade	RB-deficient GBM models resistant; RB-selected clinical trial still negative	Exclusion marker; not sufficient as sole inclusion criterion	NGS + RB IHC; baseline and on-treatment pRB	Necessary/exclusion biomarker	[11,12,13,14,58]
CDKN2A/B loss/p16 loss	Removes endogenous CDK4/6 inhibition and may increase pathway dependence	Common GBM event; prognostic/aggressive biology in survival cohorts; not predictive alone	Enrichment marker only in RB-intact tumors; interpret with broader CNV context	NGS/CNV; p16 IHC	Candidate enrichment marker	[9,10,11,41,90,120]
CDK4 amplification	Pathway activation, often in broad amplicons/co-amplified programs	Common GBM driver; may coexist with MDM2/other CNVs and trial population bias	Potential enrichment marker if RB intact and no strong bypass programs	NGS/CNV; copy-number subtype stratification	Candidate but insufficient alone	[9,10,90,120,121]
CDK6 expression/activity	May indicate functional CDK4/6 dependence in certain cell states	Proneural GSC models with high CDK6 more palbociclib-sensitive	Potential functional marker, especially with E2F/proneural state	RNA-seq, IHC, phosphoproteomics	Exploratory positive marker	[24]
CCND1/2/3 activity	Drives CDK4/6 complex formation	General CDK biology and broader sensitivity analyses support cyclin D-high states	Potential positive marker if RB intact and CCNE1/CDK2 low	RNA-seq/CNV	Exploratory	[16,123]
CCNE1/CDK2/E2F bypass	CDK4/6-independent S-phase entry	Genome-wide glioma CRISPR screen identified CCNE1 gain as a resistance modifier	Negative/resistance marker; may suggest CDK2/CHK1-type combinations	NGS/CNV + RNA-seq; E2F signature	Emerging resistance marker	[99,123]
AMBRA1 loss	Cell-cycle/checkpoint biology that modifies response	CRISPR screen identified AMBRA1 loss as a resistance modifier and CHK1 vulnerability context	Negative marker; potential combination-selection marker	NGS/RNA/protein	Emerging	[99]
PTEN/PI3K-AKT-mTOR activation	Upstream signaling can reduce CDK4/6 inhibitor sensitivity and drive escape	PTEN expression increased palbociclib sensitivity; PI3K + CDK4/6 co-inhibition synthetic lethal	PI3K-active tumors may need combination rather than monotherapy	NGS, phospho-AKT/S6, RNA signatures	Combination-selection biomarker	[17,71]
EGFR/EGFRvIII/RTK rewiring	Upstream oncogenic signaling and adaptive kinome remodeling	EGFR/CDK4 co-amplification linked to immune infiltration; EGFRvIII models show CDK6 adaptive accumulation	Combination-selection marker for EGFR/MAPK/ERK partners	NGS/CNV, EGFRvIII assay, phosphoproteomics	Context-dependent	[75,89]
tGLI1/stem-like-mesenchymal state	Transcriptional program associated with stemness and therapy resistance	tGLI1 is associated with CDK4/6 inhibitor resistance and sensitization with ketoconazole	Potential marker and combination target; investigational	RNA isoform assay; IHC if validated	Investigational	[75]
On-treatment pRB suppression/Ki-67 reduction/cell-state shift	Dynamic proof of target engagement and functional response	Phase 0 ribociclib and ribociclib/mTOR studies show tumor PD and single-cell state changes	Best early-phase go/no-go biomarker strategy	Pre/post-op tissue; IHC; snRNA-seq where feasible	Dynamic PD biomarker	[14,57,58]

## Data Availability

No new data were created or analyzed in this study. Data sharing is not applicable to this article.
